# Mutational analysis of the alpha subunit of eIF2B provides insights into the role of eIF2B bodies in translational control and VWM disease

**DOI:** 10.1074/jbc.RA120.014956

**Published:** 2021-01-30

**Authors:** Karl Norris, Rachel E. Hodgson, Tawni Dornelles, K. Elizabeth Allen, Ben M. Abell, Mark P. Ashe, Susan G. Campbell

**Affiliations:** 1 Biomolecular Sciences Research Centre, Sheffield Hallam University, Sheffield, UK; 2 Division of Molecular and Cellular Function, Faculty of Biology, Medicine and Health, The University of Manchester, Manchester, UK

**Keywords:** eIF2B, eIF2, translation initiation, VWM, eIF2, eukaryotic initiation factor 2, eIF2B, eukaryotic initiation factor 2B, FRAP, fluorescence recovery after photobleaching, GEF, guanine nucleotide exchange factor, ISR, integrated stress response, NIH, National Institutes of Health, SCD, synthetic complete media, VWM, vanishing white matter

## Abstract

Eukaryotic initiation factor 2B (eIF2B) serves as a vital control point within protein synthesis and regulates translation initiation in response to cellular stress. Mutations within eIF2B result in the fatal disease, leukoencephalopathy with vanishing white matter (VWM). Previous biochemical studies on VWM mutations have illustrated that changes in the activity of eIF2B poorly correlate with disease severity. This suggests that there may be additional characteristics of eIF2B contributing to VWM pathogenesis. Here, we investigated whether the localization of eIF2B to eIF2B bodies was integral for function and whether this localization could provide insight into the pathogenesis of VWM. We demonstrate that the regulatory subunit, eIF2Bα, is required for the assembly of eIF2B bodies in yeast and that loss of eIF2B bodies correlates with an inability of cells to regulate eIF2B activity. Mutational analysis of eIF2Bα showed that missense mutations that disrupt the regulation of eIF2B similarly disrupt the assembly of eIF2B bodies. In contrast, when eIF2Bα mutations that impact the catalytic activity of eIF2B were analyzed, eIF2B bodies were absent and instead eIF2B localized to small foci, termed microfoci. Fluorescence recovery after photobleaching analysis highlighted that within these microfoci, eIF2 shuttles more slowly indicating that formation of eIF2B bodies correlates with full eIF2B activity. When eIF2Bα VWM mutations were analyzed, a diverse impact on localization was observed, which did not seem to correlate with eIF2B activity. These findings provide key insights into how the eIF2B body assembles and suggest that the body is a fundamental part of the translational regulation *via* eIF2α phosphorylation.

Eukaryotic genomes encode many thousands of proteins, and through the process of protein synthesis from mRNA or translation, the cell can rapidly control its gene expression profile to promote cellular homeostasis. The initiation step of translation is rate limiting and therefore provides a critical control point in gene expression. The highly conserved heterotrimeric G-protein, eukaryotic initiation factor 2 (eIF2), is essential for the initiation and regulation of translation. In its active GTP bound form, eIF2 binds to a methionyl initiator tRNA molecule to form a ternary complex (1). Facilitated by a number of eIFs, the ternary complex is loaded onto the 40S ribosomal subunit and recruited to a target mRNA molecule, allowing for subsequent ribosomal scanning and start codon recognition (1). Upon start codon recognition, eIF2–GTP is hydrolyzed by the GTPase-activating protein, eIF5 (2), and is released from the ribosome in its inactive GDP bound form, in complex with eIF5 (1). For subsequent rounds of translation to occur within the cell, active eIF2–GTP must be replenished. eIF2 has a higher affinity for GDP than GTP (3), and therefore, the multisubunit protein eIF2B is required to catalyze this guanine nucleotide exchange (4). In yeast, eIF5 functions as a GDP dissociation inhibitor, preventing any spontaneous recycling of GDP to GTP on eIF2 (5). In addition to its role as a guanine nucleotide exchange factor (GEF), in yeast, eIF2B also acts as a GDP dissociation inhibitor displacement factor, releasing eIF2–GDP from eIF5 (6).

Although functionally similar to other GEFs of the Ras superfamily, eIF2B has a much more complex quaternary structure. It is composed of five nonidentical subunits termed, α, β, δ, γ, and ε, encoded in yeast by the genes: *GCN3*, *GCD7*, *GCD2*, *GCD1*, and *GCD6*, respectively. The native form of eIF2B is composed of a dimer of heteropentamers and so is decameric (7, 8). eIF2Bγ–eIF2Bε heterodimers reside on both flanks of the structure and are responsible for the protein's GEF activity (4). Heterodimers of eIF2Bβ and δ subunits bind eIF2Bγε heterodimers and reside in the core of the decameric arrangement, stabilized in this conformation by an eIF2Bα homodimer. Structural analysis of both yeast and mammalian eIF2B has provided models for how the decameric structure is formed and how eIF2 can interact with the decamer (9, 10, 11, 12, 13). In mammalian cells, Wortham *et al.* (14) identified that all eIF2B subunits, except eIF2Bα, are stoichiometrically regulated. Stable expression of eIF2Bε relies on similar levels of γ to be coexpressed; correspondingly, eIF2Bδ requires similar levels of eIF2Bεγ and β. Any surplus protein subunits are degraded by the ubiquitin–proteasome system. This study indicated that the eIF2B holocomplex may be built around the eIF2Bεγβδ subcomplex, with eIF2Bα_2_ homodimers bridging two tetramers to complete the decameric holocomplex. In line with this model, Tsai *et al.* (15) demonstrated that in the absence of eIF2Bα, eIF2B exists as an eIF2Bεγβδ tetramer.

The subunit complexity of eIF2B lends itself as a target of tight regulation. As the process of translation involves a significant amount of cellular energy, tight regulation is crucial in response to adverse cellular conditions. One of the best studied and most diverse mechanisms of translational control in response to cellular stress is the integrated stress response (ISR), known as the general amino acid control pathway in yeast (16, 17). The ISR involves a series of cellular stress–sensing pathways that regulate translation through the common mechanism of eIF2 phosphorylation (18). In mammalian cells, four eIF2α kinases exist, whereas in yeast, a single kinase, general control nonderepressible 2, is responsible for the phosphorylation of eIF2α at serine 51 in response to amino acid starvation (19). Phosphorylation of eIF2 at this site converts eIF2 from a substrate to an inhibitor of eIF2B GEF activity. While the α, β, and δ subunits of eIF2B are dispensable for GEF activity, they are responsible for tight regulation of this activity by phosphorylated eIF2 (20, 21). The inhibition of eIF2B GEF activity induces global translational repression within the cell. Paradoxically, a number of stress-responsive proteins are translationally upregulated to favor homeostatic reprogramming (18). The translation of these proteins is most commonly controlled by the presence of upstream open reading frames in the 5'UTR of the mRNAs, first demonstrated in yeast for *GCN*4 mRNA (22).

Recent structural studies in both yeast and mammalian systems have solved structures of eIF2B bound to both phosphorylated and nonphosphorylated eIF2α (9, 10, 11, 12, 13, 23). Interestingly, although the structure of eIF2B is highly conserved across species (9, 10, 13), phosphorylated eIF2α appears to interact and inhibit eIF2B *via* distinct mechanisms within yeast and mammalian cells. In mammalian cells, phosphorylated and nonphosphorylated eIF2α bind to different regions of eIF2B, whereas in yeast, they share a binding pocket (10). eIF2α binds to the eIF2B regulatory subunits, and this binding position is favorable for nucleotide exchange. Upon phosphorylation of eIF2α, conformational changes in its structure are believed to enhance the binding of eIF2α to eIF2Bα and δ subunits. It is this conformational change in eIF2B that displaces the catalytic domain of eIF2Bε, responsible for carrying out nucleotide exchange, from its original close proximity to eIF2, thus inhibiting nucleotide exchange (10).

Localization studies in both yeast and mammalian systems have shown that eIF2B accumulates at specific foci within the cytoplasm of the cell (24, 25, 26, 27, 28, 29, 30, 31). These foci have been termed eIF2B bodies and in yeast appear as one large cytoplasmic granule that morphologically exists as a filamentous-like structure. eIF2 also localizes to eIF2B bodies raising the possibility that eIF2B bodies are sites where eIF2B GEF activity occurs and is regulated within the cell. In 2005, Campbell *et al.* (24) demonstrated that in *Saccharomyces cerevisiae* eIF2B is a stable component of eIF2B bodies, whereas the association of eIF2 is dynamic, with eIF2 transiting through the eIF2B body at a rate that correlates to the cellular level of eIF2B GEF activity. In 2010, Taylor *et al.* (26) observed that eIF2B bodies were motile throughout the cytoplasm. This movement is important for effective translation initiation as butanol treatment hinders eIF2B body movement, and this lack of movement correlates with the inhibition of translation initiation. These data provide evidence to suggest eIF2B bodies are sites of eIF2B GEF activity and regulation; however, recent interest in eIF2B bodies has provided some conflicting interpretations. One study has suggested that eIF2B bodies are not present in *S. cerevisiae* under steady-state growth, but only form under glucose-limiting conditions (28). Interestingly, another study highlighted that eIF2B bodies were not induced during acute glucose starvation but were formed upon energy depletion (during stationary phase) as a mechanism for sequestering eIF2B proteins to inhibit their function (30, 31). Therefore, the significance of eIF2B assembly into eIF2B bodies for its function and regulation remains unclear.

The importance of understanding eIF2B localization is heightened by the fact that in mammalian cells, mutations within eIF2B result in the fatal and autosomal recessive disease, leukoencephalopathy with vanishing white matter (VWM) (32). To date, over 200 VWM mutants have been identified spanning all five subunits (33). The relationship between mutant eIF2B function and disease severity remains poor. VWM-causing mutations have been identified that affect neither decameric complex formation nor eIF2B activity *in vitro* but cause some of the most severe forms of VWM *in vivo* (34, 35). Understanding eIF2B body formation and regulation could uncover common pathophysiological mechanisms across the broad spectrum of causative mutations. Although *GCN3*, which encodes eIF2Bα, is the only nonessential *eIF2B* gene in yeast, it is still critical for stabilizing eIF2B in its decameric conformation and for the regulation of eIF2B activity during cellular stress. Here, we investigate the importance of eIF2Bα to eIF2B body assembly and activity. Using *S. cerevisiae*, we show that eIF2Bα (Gcn3p in *S. cerevisiae*) is central to the formation of eIF2B bodies and suggest that eIF2B bodies are a fundamental part of the translational regulation *via* eIF2α phosphorylation. In addition, VWM-causative mutations disrupt eIF2B body formation and regulation, providing the first evidence that eIF2B localization is altered by VWM-causing mutations.

## Results

### eIF2B localization varies between different yeast strains

**Figure 1:**
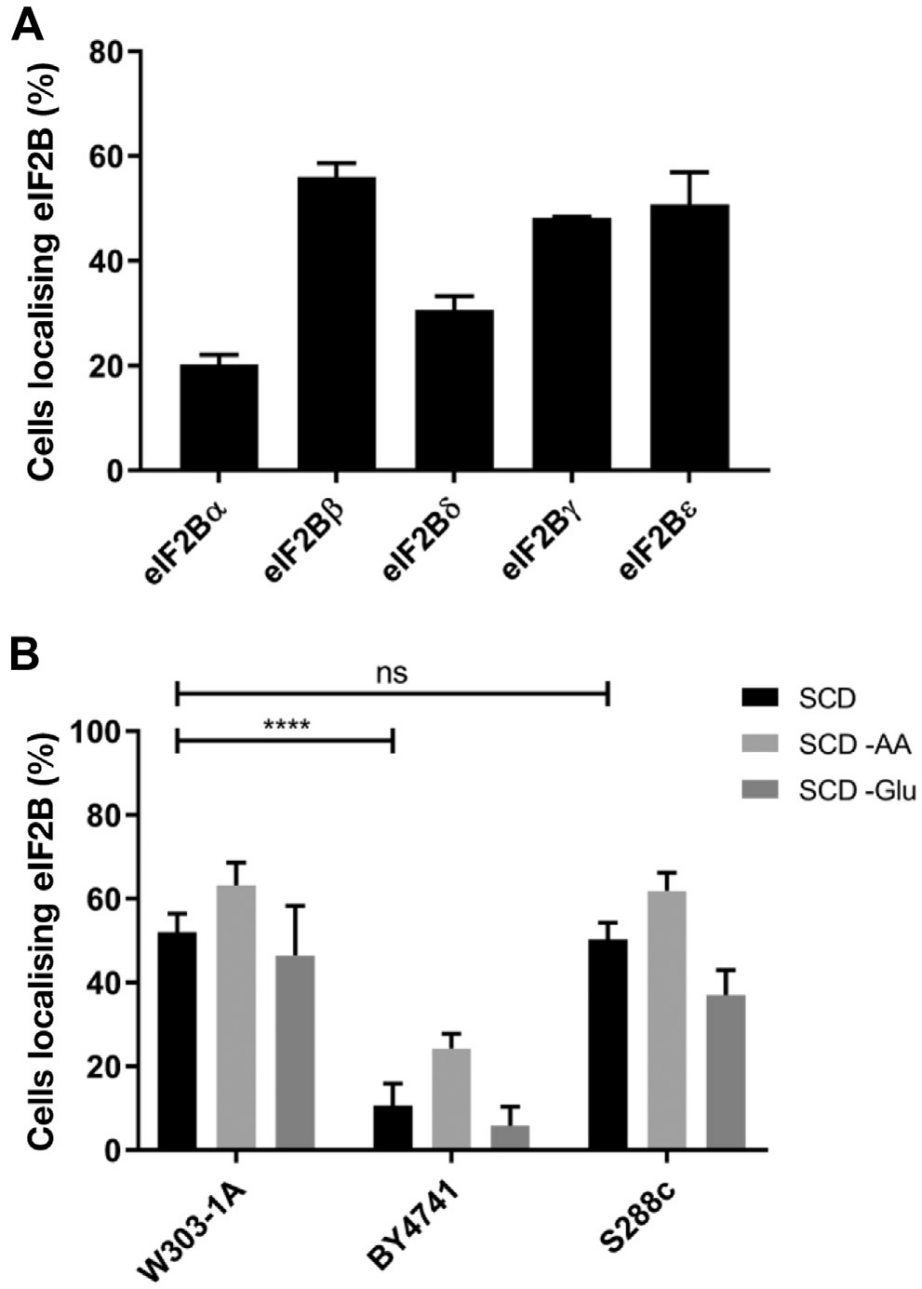
**The percentage of cells containing eIF2B bodies differs depending on the subunit C-terminally tagged and the lab strain of *Saccharomyces cerevisiae*.***A*, each eIF2B subunit was individually C-terminally GFP tagged in the W303-1A background strain. The number of cells displaying eIF2B bodies was analyzed and presented as a percentage of total cells counted. A minimum of 50 cells were counted for each experiment, *n* = 3. *B*, *GCD1* (eIF2Bγ) was C-terminally GFP tagged in the *S. cerevisiae* background strains W303-1A (yMK880), BY4741 (ySC9), and S288c (yMK1180). Following logarithmic growth, each strain was subjected to amino acid and glucose starvation for 30 min, before eIF2B localization was assessed. The number of cells displaying eIF2B bodies was analyzed and presented as a percent of total cells counted. A minimum of 50 cells were counted for each experiment, *n* = 3. ∗∗∗∗*p* < 0.0001. eIF2B, eukaryotic initiation factor 2B; ns, not significant; SCD, synthetic complete media.

Using C-terminal yeGFP-tagged eIF2B subunits, we have previously shown that, during steady-state growth, all five subunits of eIF2B colocalize to eIF2B bodies (24, 25, 26, 27). Recently, a number of groups have presented conflicting data about whether these eIF2B bodies exist during steady-state growth or only form under specific starvation conditions (28, 30, 31). A potential explanation for these conflicting results is that different GFP tags may be influencing the aggregation of eIF2B. In order to investigate this, we first observed eIF2B body formation by individually tagging each eIF2B subunit with GFP and determining the percentage of cells in which eIF2B bodies were present. We hypothesized that if the eIF2B bodies that we have observed during steady-state growth were due to aggregation of the GFP tag, we would expect to observe a similar percentage of cells containing eIF2B bodies for all five GFP-tagged eIF2B subunits. A similar percentage of cells contained eIF2B bodies when the eIF2Bγ (48%), ε (51%), and β (56%) subunits were tagged, whereas a lower percentage of cells contained eIF2B bodies when eIF2Bα (20%) or δ (30%) subunits where tagged (Fig. 1*A*). These results suggest that it is unlikely that the GFP tag we have used is responsible for eIF2B body formation; rather, the ability of eIF2B to form bodies is influenced by the subunit that is tagged.

Another possible explanation for differences in eIF2B body formation is variation between yeast strains, which are known to have differing responses to environmental stresses (36). In our previous studies, we have utilized *S. cerevisiae* W303-1A strain, whereas others have characterized eIF2B bodies in the BY4741 strain. To determine whether various laboratory strains localize eIF2B differently, the eIF2Bγ subunit was C terminally GFP tagged in the auxotrophic W303-1A, BY4741, and S288c background strains, and eIF2B localization was assessed. There was no difference in growth between strains (Fig. S1). During steady-state growth, a similar number of cells showing eIF2B bodies were observed for the W303-1A and S288c backgrounds (52% and 50%, respectively), whereas a significantly lower number of cells were observed to show eIF2B bodies in the BY4741 strain background (11%).

To determine whether there was any change in the localization upon stress, the cells were subjected to acute glucose starvation (30 min) and amino acid starvation (15 min). Previously, we had observed that the level of fluorescence within eIF2B bodies increased upon amino acid starvation and that this was dependent on the phosphorylation of eIF2α (24). Interestingly, upon amino acid starvation, an increase in the number of cells showing eIF2B bodies was observed for all strains (Fig. 1*B*). For the W303-1A and S288c strains, this increased percentage resulted in a 1.2-fold increase of cells with eIF2B bodies, whereas for the BY4741 strain, this increased percentage of cells represents a much greater 2.3-fold increase. In contrast to these increases, following glucose starvation, the number of cells displaying eIF2B bodies slightly decreased for all strains W303-1A, eIF2Bγ–GFP (46%), S288c, eIF2Bγ–GFP (37%), and BY4741 eIF2Bγ–GFP (6%); however, compared with synthetic complete media (SCD), these differences were not found to be statistically significant. These results are consistent with data showing that eIF2B is not directly involved in translational control following glucose starvation (37, 38, 39). Overall, these results suggest that under normal growth, the level of eIF2B localization to bodies depends on the *S. cerevisiae* strain, but that the trends in terms of responses to nutritional stress are similar and are dependent on stresses that target eIF2B activity.

### Deletion of eIF2Bα disperses eIF2B bodies

**Figure 2:**
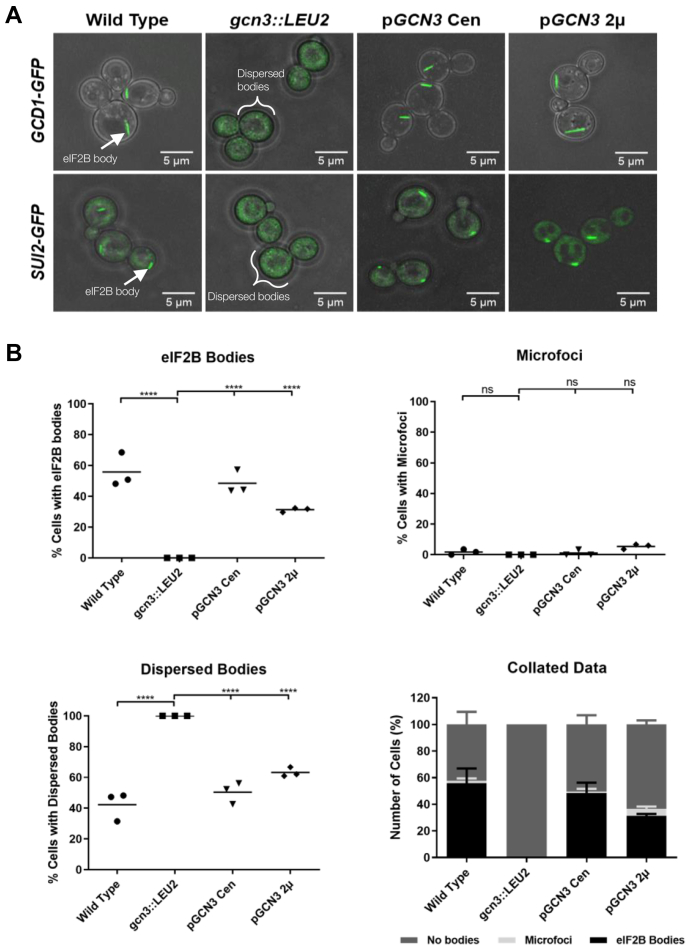
**eIF2Bα is required for eIF2B body formation.***A*, confocal microscopy of *GCD1-yeGFP* (yMK880), *SUI2-yeGFP* (yMK883), *GCD1-yeGFPgcn3::LEU2* (yMK1402), and *SUI2-yeGFPgcn3::LEU2* (ySC16). Null strains were transformed with a low-copy Cen plasmid (pAV1170) or a high-copy 2μ plasmid (pAV1117) containing WT*GCN3*. *B*, a minimum of 100 cells were counted and assessed as to whether eIF2B bodies were present, dispersed, or localized to microfoci. Overnight cultures were diluted in synthetic complete media to 0.2 absorbance at 600 nm and incubated at 30 °C with shaking until exponential growth was reached. *n* = 3. Error bars are representative of SD. ∗*p* < 0.05, ∗∗*p* < 0.01, ∗∗∗*p* < 0.001, ∗∗∗∗*p* < 0.0001. eIF2B, eukaryotic initiation factor 2B; ns, not significant.

In our previous studies, we have shown that strains harboring *gcn3* point mutations show no eIF2B bodies (26). eIF2Bα (Gcn3p in yeast) is essential for decameric formation, and therefore, this phenotype could reflect destabilization of the decameric complex. eIF2Bα is dispensable for eIF2B GEF activity but is required to regulate this activity in response to cellular stress. Within the eIF2B decameric complex, the eIF2Bα homodimer forms part of the regulatory core, which after cellular stress forms a high-affinity interaction with phosphorylated eIF2 to prevent GDP to GTP exchange on nonphosphorylated eIF2 within the cell (23). Strains deleted for the *eIF2Bα* gene are viable in yeast under steady-state conditions; however, they cannot survive ISR-activating stress conditions (18). To test whether eIF2B bodies form when it is no longer possible to stabilize the decameric complex, eIF2Bα (Gcn3p in yeast) was deleted in strains harboring either C-terminal GFP-tagged eIF2Bγ (Gcd1p in yeast) or eIF2α (Sui2p in yeast). Upon deletion of the *eIF2Bα* gene, eIF2B does not localize to eIF2B bodies and instead is found completely dispersed throughout the cytoplasm as visualized using either eIF2Bγ–GFP and eIF2α–GFP strains (Fig. 2*A*). The lack of response to conditions causing eIF2α phosphorylation in the gcn3 null strain was also confirmed *via* polysome profiling (Fig. S2*A*). Localization of eIF2B to eIF2B bodies was rescued when eIF2Bα (Gcn3p) was exogenously expressed on either a low-copy centromeric or a high-copy 2 micron plasmid (Fig. 2*A* and Fig. S2). Interestingly, a slight decrease in the number of cells showing eIF2B bodies was observed when eIF2Bα was overexpressed (50% ± 9.0% *versus* 37% ± 1.2%) (Fig. 2*B*). Intriguingly, in addition to eIF2B bodies, for all strains except the *gcn3* null strain, eIF2B also localized to multiple smaller punctate foci, which we termed microfoci; however, during steady-state growth, such microfoci were rarely observed (Fig. 2*B*).

These results show that eIF2Bα is required for the localization of eIF2B to eIF2B bodies and are suggestive that the complete decameric complex may be required for eIF2B body assembly.

### Mutations in eIF2Bα alter the localization of eIF2B

**Figure 3:**
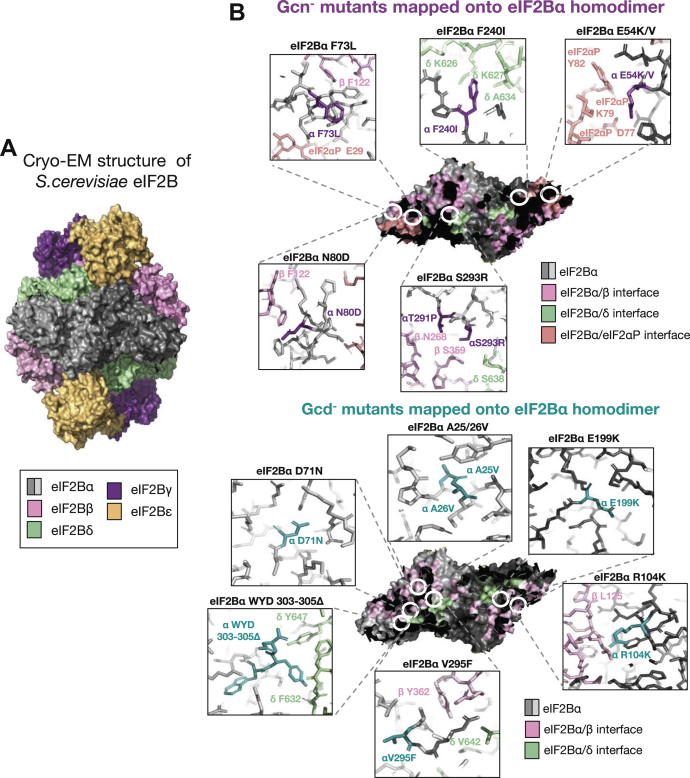
**Structural schematic highlighting eIF2Bα mutations.***A*, the cryo-EM structure for *Sacccharomyces cerevisiae* eIF2B is shown in ribbon representation. *B*, the eIF2B homodimer has been enlarged and is shown as a surface representation with interfaces between eIF2Bα and the other eIF2B/eIF2 subunits color coded. The position of the amino acid mutations introduced in this study is highlighted. These mutants were modeled on the cryo-EM structure data, and *stick* representations are shown with amino acids from neighboring eIF2B/eIF2 subunits highlighted. Gcn^−^ mutants are shown in *purple* and Gcd^−^ mutants in *turquoise*. These mutations were expressed in the yMK1402 background strain. (Gcn^−^ mutants Protein Data Bank ID: 6i3m and Gcd^−^ mutants Protein Data Bank ID: 6i7t). eIF2B, eukaryotic initiation factor 2B.

To further investigate the role of eIF2Bα in the localization of eIF2B and to determine whether eIF2B bodies have a functional role in regulating eIF2B activity, a series of well-characterized Gcn3p mutants were examined (Fig. 3) (40). These mutations confer two distinct phenotypes that affect either the regulatory (Gcn^−^) or catalytic (Gcd^−^) activity of eIF2B. Gcn^−^ mutations prevent eIF2B activity from responding to cellular stress by impeding eIF2B–eIF2α-P interactions; therefore, allowing cells to continue eIF2B exchange activity even in the presence of phosphorylated eIF2ɑ (Fig. S3*A*). Gcd^−^ mutations reduce eIF2B GEF activity, which constitutively induces the expression of the stress responsive transcription factor Gcn4p (Fig. S3*B*).

**Figure 4:**
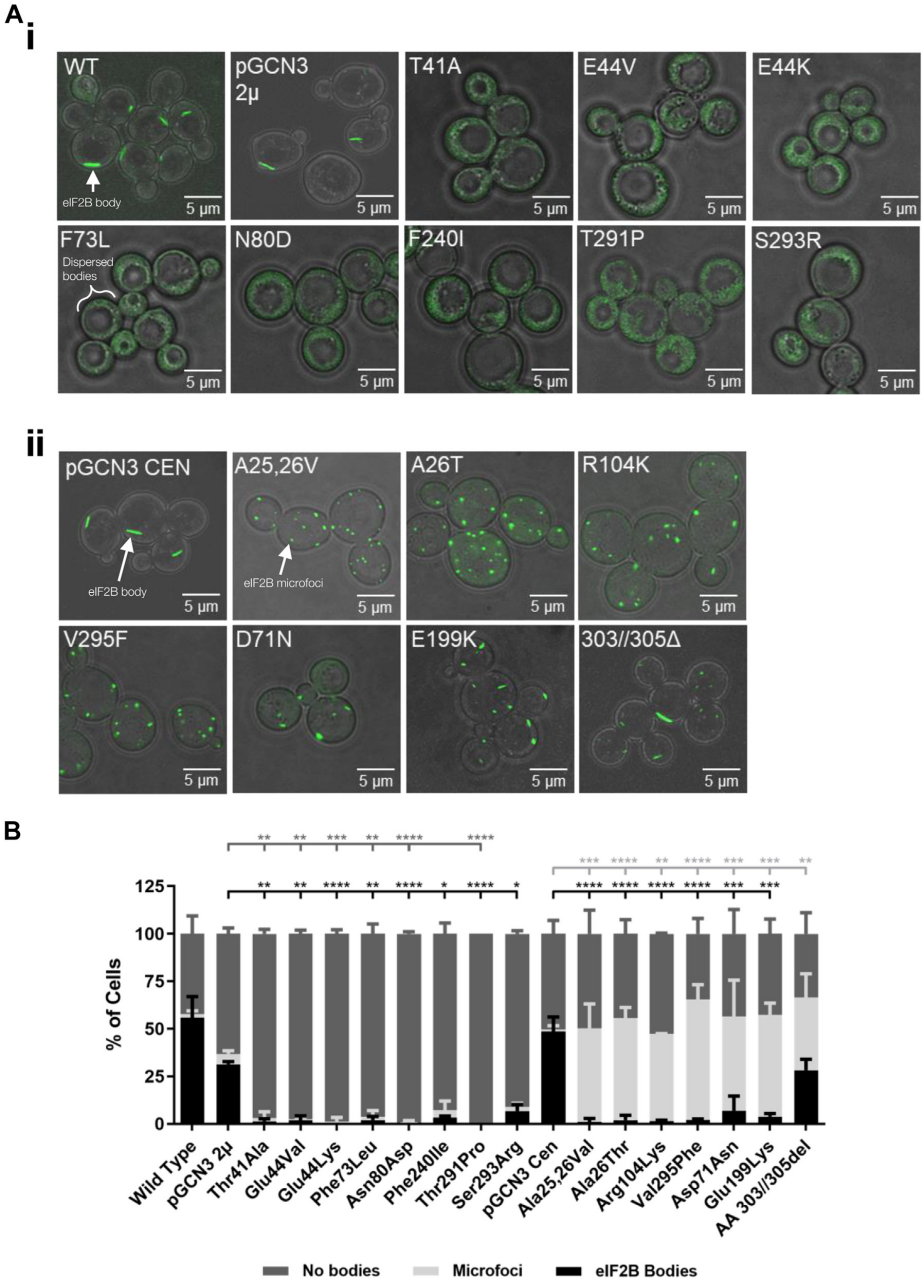
**eIF2Bɑ mutants alter the localization of eIF2B bodies.***A*, cells were grown to log phase, and confocal microscopy was used to image the strain yMK1402 (*GCD1-yeGFPgcn3::LEU2*) containing a series of (*i*) Gcn^−^ mutants (pAV1108–13, 15, and 16) and (*ii*) Gcd^−^ mutants (pAV1238–44 and 68). *B*, cells from each strain were counted to assess whether eIF2B bodies were present, dispersed, or localized to microfoci. The first part of the graph corresponds to the gcn^−^ mutants, and the second part shows the gcd^−^ mutants. For each strain analyzed, the localization of eIF2B was assessed for 100 cells per biological replicate, *n* = 3. Error bars are representative of SD. ∗*p* < 0.05, ∗∗*p* < 0.01, ∗∗∗*p* < 0.001, ∗∗∗∗*p* < 0.0001. eIF2B, eukaryotic initiation factor 2B; ns, not significant.

Eight Gcn^−^ and seven Gcd^−^ Gcn3p mutants were analyzed for their impact on eIF2B body formation. The position of these mutations within the structure of Gcn3p can be seen in Figure 3. For all mutants analyzed, eIF2B body formation was affected (Fig. 4*A*). Interestingly, for the regulatory mutants (Gcn^−^), no eIF2B bodies were observed, with the exception of the Gcn3 (S293R) mutant, where a small proportion of cells showed eIF2B bodies (6.7%) (Fig. 4, *Ai*–*B*). This loss of eIF2B bodies was not because of a decrease in protein expression (Fig. S2*B*). This dispersal of the eIF2B body localization observed in the Gcn3p regulatory mutants resembles the dispersal of eIF2B bodies in the gcn3 null strain and therefore seems likely to reflect the inability of these mutants to regulate eIF2B GEF activity rather than any change in subunit levels (Fig. 2*A*).

While the regulatory mutants dispersed the localization of eIF2B completely, the catalytic mutants resulted in the localization of eIF2B to multiple smaller and punctate foci, termed microfoci (Fig. 4*Aii*). These mutants also resulted in moderate reductions of subunit expression (Fig. S2*B*). The only exception within this group of catalytic mutations was the AA 303//305Δ mutant, where similar numbers of cells either dispersed eIF2B (33.5%), formed microfoci (38.3%), or formed eIF2B bodies (28.2%) (Fig. 4*B*).

### FRAP analysis identifies slower exchange within the microfoci

Using fluorescence recovery after photobleaching (FRAP) analysis, we have previously demonstrated that the GEF activity of eIF2B is controlled and regulated in eIF2B bodies, as the rate of shuttling of eIF2 through eIF2B bodies was perturbed when phosphorylated eIF2ɑ was present (24, 26). Since the eIF2Bα catalytic mutants display a Gcd phenotype as assessed by their increased Gcn4p levels during steady state (Fig. S3*B*), we postulated that disruption of the large eIF2B body to give microfoci (Fig. 4*Aii*) may decrease the shuttling of eIF2 through eIF2B. To test this hypothesis, FRAP analysis was carried out on the AA25, 26VV, E199K, and AA 303//305Δ mutant strains. As the mutant AA 303//305Δ also formed eIF2B bodies as well as microfoci, this catalytic mutant represented a unique opportunity to investigate differences in the shuttling of eIF2 between the two types of eIF2B localization in a single cell.

**Figure 5:**
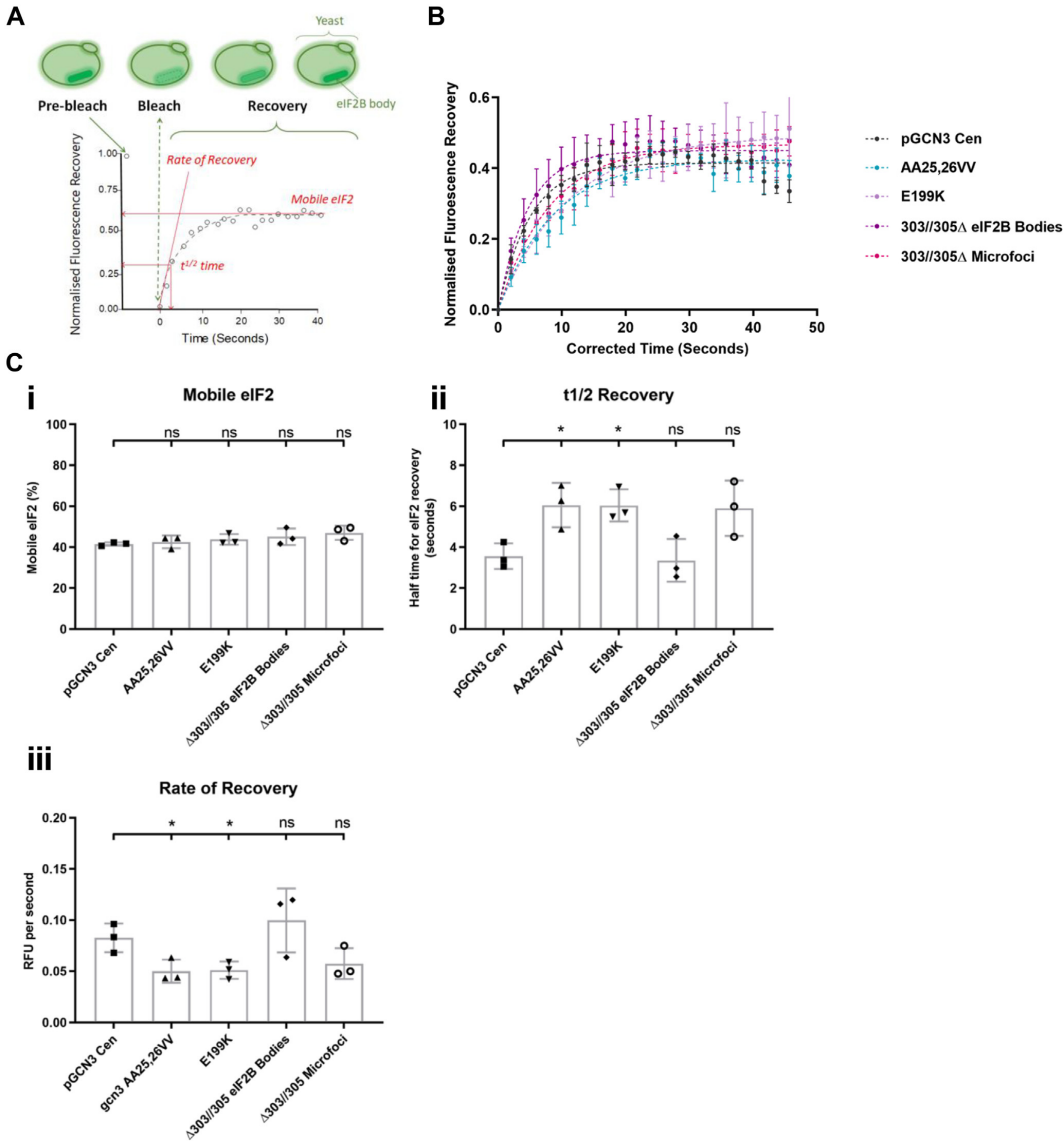
**Fluorescence recovery after photobleaching (FRAP) analysis of gcn3 Gcd**^**−**^**mutations reveal slower shuttling of eIF2 through eIF2B microfoci.***A*, schematic representation of FRAP technique. The eIF2B body/microbody is bleached, and the intensity of fluorescent signal within this region is measured, normalized against intensity of the prebleached signal, and plotted against time. Mobile eIF2 is calculated as the plateau of the FRAP curve. t1/2 Recovery is calculated as the fluorescence measured at the half time point of total recovery. The rate or recovery is calculated by dividing the plateau value by the rate constant (*k*). *B*, three gcn3 Gcd^−^ mutations AA25,26VV (pAV1239), E199K (pAV1244), and 303//305Δ (pAV1268) as well as the GCN3 Cen plasmid (pAV1170) were exogenously expressed in SUI2-yeGFPgcn3::LEU2(ySC16) to measure eIF2 recovery within eIF2B bodies. Both eIF2B body and microfoci were analyzed for the 303//305Δ mutant. *B*, normalized FRAP recovery curves Gcd^−^ mutations AA25,26VV (pAV1239), E199K (pAV1244), and 303//305Δ (pAV1268) as well as the GCN3 Cen plasmid (pAV1170) were exogenously expressed in SUI2-yeGFPgcn3::LEU2(ySC16) *C*, (*i*) bar chart representing the mobile eIF2 within the foci as a percentage; (*ii*) bar chart representative of half the time needed for eIF2 to fully recover; (*iii*) bar chart depicting the rate of eIF2 recovery. Data are representative of 25 cells, *n* = 3. Error bars are representative of SD. ∗*p* < 0.05, ∗∗*p* < 0.01, ∗∗∗*p* < 0.001, ∗∗∗∗*p* < 0.0001. eIF2B, eukaryotic initiation factor 2B; ns, not significant.

Fluorescence recovery of eIF2ɑ–GFP was measured over time, and mean recovery curves are shown in Figure 5*B*. Representative images across the different stages of the FRAP experiment are shown in Figure S4. Although the percentage of mobile eIF2 did not deviate significantly between the wildtype and any of the catalytic mutants (Fig. 5*Ci*), the rate of eIF2 recovery into eIF2B bodies after photobleaching was consistently slower in the catalytic mutants than in wildtype cells (Fig. 5*Ciii*). This is consistent with our previous analysis, where we showed a similar decreased rate of eIF2 shuttling for catalytic mutants in eIF2Bε (24). Consistent with this decreased rate, the AA25,26VV and E199K mutants exhibited increased half time for eIF2 recovery in comparison to plasmid-borne wildtype *GCN3* control (Fig. 5*Cii*). Intriguingly, eIF2 shuttling through eIF2B bodies within the AA 303//305Δ mutant was comparable to wildtype, whereas, eIF2 shuttling through microfoci within this same mutant declined similarly to the other microfoci-forming mutants AA25,25VV and E199K (Fig. 5). Therefore, FRAP analyses of eIF2 suggest that the formation of eIF2B bodies enhances the eIF2 shuttling capacity relative to eIF2B microfoci.

### VWM mutations in the α subunit of eIF2B result in altered eIF2B localization

Mutations within eIF2B result in the fatal disease leukoencephalopathy with VWM. Previous biochemical studies on VWM mutations have relied upon *in vitro* analysis of eIF2B activity, complex integrity, and protein stability (33).

While these analyses have illustrated that VWM mutations can impact the activity of eIF2B, the scale of observed effects correlates poorly with disease severity (34). Given the results from our localization studies for mutations that differentially impact the regulatory or catalytic activity of eIF2B, it is possible that VWM mutations may also affect the integrity and GEF activity of eIF2B bodies; providing mechanistic insights into VWM pathophysiology. VWM-causative mutations have been characterized in all five subunits of eIF2B, with eight mutations identified in eIF2Bα (32, 33, 41, 42).

**Figure 6:**
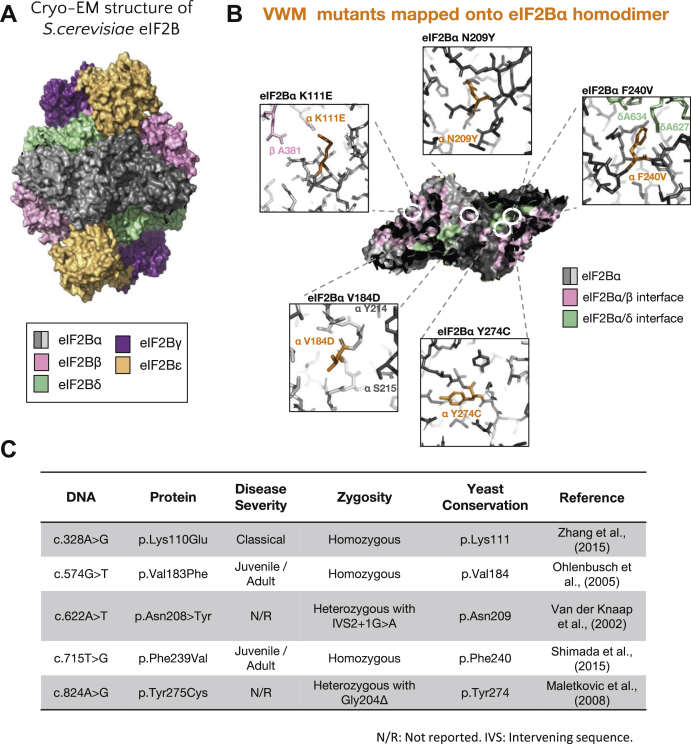
**Structural schematic highlighting eIF2Bα VWM mutations.***A*, the cryo-EM structure for *Saccharomyces cerevisiae* eIF2B is shown on the *left* in surface representation. *B*, on the *right*, the eIF2B homodimer has been enlarged and is shown as a surface representation with interfaces between eIF2Bα and the other eIF2B subunits color coded. The position of the amino acid mutations introduced in this study is highlighted. These mutants were modeled on the cryo-EM structure data and are shown in *orange* as stick representations, with amino acids from neighboring eIF2B subunits highlighted. *C*, the VWM disease severity, zygosity, and yeast conservation of these mutations are outlined. These mutations were expressed in the ySC16 background strain. (Protein Data Bank ID: 6i7t). eIF2B, eukaryotic initiation factor 2B; VWM, vanishing white matter.

**Figure 7:**
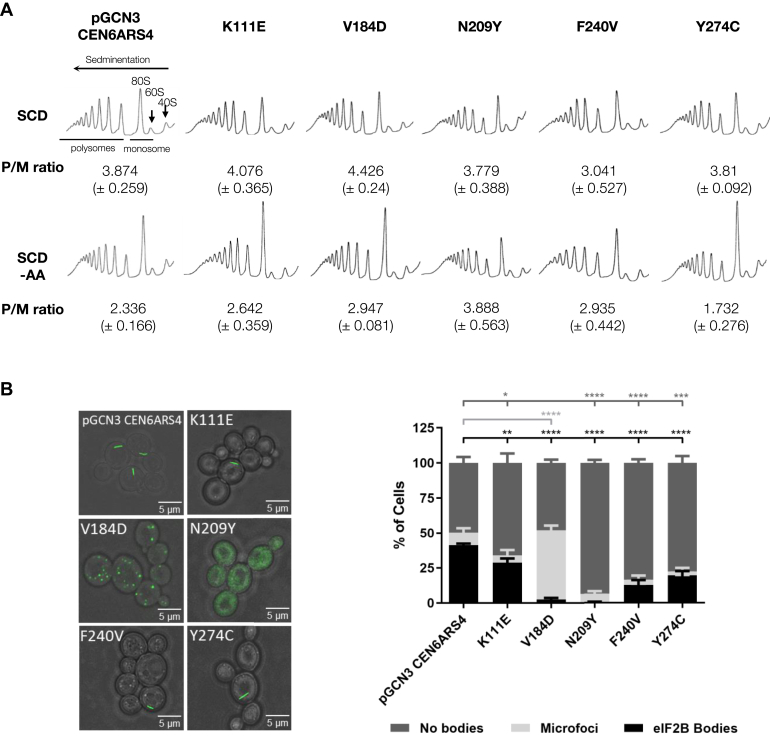
**eIF2B localization in the presence of gcn3-containing vanishing whie matter (VWM) missense mutations.***A*, polysome analysis of the strain yMK1402 (*GCD1-yeGFPgcn3::LEU2*) expressing gcn3 VWM mutants and the low-copy WTGCN3 plasmid (ySC91–96). Polysome analysis was as described in Experimental procedures. Polysome/monosome ratios were calculated from measuring the area under the polysome peaks and dividing by the monosome peak area. *B*, (*i*) cells were grown to log phase, and confocal microscopy was used to image the strain yMK1402 (*GCD1-yeGFPgcn3::LEU2*) expressing gcn3 VWM mutants, Gcn3p^K11E^ (*p[GCN3K111EURA3CEN6ARS4]*), Gcn3p^V184D^ (*p[GCN3V184DURA3CEN6ARS4]*), Gcn3p^N209Y^ (*p[GCN3N209YURA3CEN6ARS4]*), Gcn3p^F230V^ (*p[GCN3F240VURA3CEN6ARS4]*), Gcn3p^Y274C^ (*p[GCN3Y274CURA3CEN6ARS4]*), and the low-copy WT plasmid *p[GCN3URA3CEN6ARS4]*. (*ii*) cells from each strain were counted to assess whether eIF2B bodies were present, dispersed, or localized to microfoci. For each strain analyzed, the localization of eIF2B was assessed for 100 cells per replicate, *n* = 3. Error bars are representative of SD. ∗*p* < 0.05, ∗∗*p* < 0.01, ∗∗∗*p* < 0.001, ∗∗∗∗*p* < 0.0001. ns, not significant; SCD, synthetic complete media.

Of the eight mutations identified in eIF2Bɑ, one results in a frameshift, one results in a deletion, and six are missense mutations. Of the six missense-mutated residues, only one is not conserved in yeast, P278R (Q277 in yeast). Therefore, five VWM missense mutations, K111E, N209Y, V184D, F240V, and Y274C, were investigated (Fig. 6). While these mutations cause a range of disease severities in humans, no change in yeast cell growth was observed (Fig. S5*A*). To determine what impact the VWM mutants had on the control of translation initiation, exponential cultures of the various strains were subjected to amino acid starvation, and polysome profiling was performed (Fig. 7*A*). In line with previous work, N209Y was identified as a Gcn^−^ mutation and was unable to respond to stress (43). While the VWM mutation F240V displayed a similar phenotype to N209Y, the remaining mutants, V184D, K111E, and Y274C, were all able to respond to amino acid starvation in a similar manner to the control strain (Fig. 7*A*). We next determined whether these VWM eIF2Bα mutations had any impact on the localization of eIF2B to eIF2B bodies. We hypothesized that if the mutations N209Y and F240V had Gcn^−^ phenotypes that caused a disruption to the regulatory role of Gcn3p, then eIF2B bodies would be dispersed throughout the cytoplasm. This was the case when eIF2B localization was observed in the presence of the eIF2Bα N209Y mutation (Fig. 7*B*). In contrast, for the eIF2Bα F240V mutant strain, which displayed a Gcn^−^ phenotype and could not respond to amino acid starvation, eIF2B bodies were partially disrupted with a decreased percentage of cells displaying them (13%) (Fig. 7*B*). The three mutations that do not affect the regulatory function of eIF2Bα either decreased the number of cells displaying eIF2B bodies (K111E [29%] and Y274C [20%]) or in the case of the V184D mutant formed microfoci (Fig. 7*B*). Surprisingly, these mutations did not show decreased eIF2B activity as measured by induced Gcn4 expression (Fig. S5*B*).

We next made use of our FRAP assay to determine if these mutants impacted upon the ability of eIF2α to shuttle through eIF2B bodies. Intriguingly, although eIF2B bodies were present within the mutants V184D and F240V, eIF2α–GFP did not localize to discrete cytoplasmic bodies in these mutants and instead was dispersed throughout the cytoplasm (Fig. S5*D*). Therefore, an analysis of eIF2 shuttling was not possible in these mutants, and the lack of colocalization of eIF2 and eIF2B may reflect differences in affinities of these complexes.

**Figure 8:**
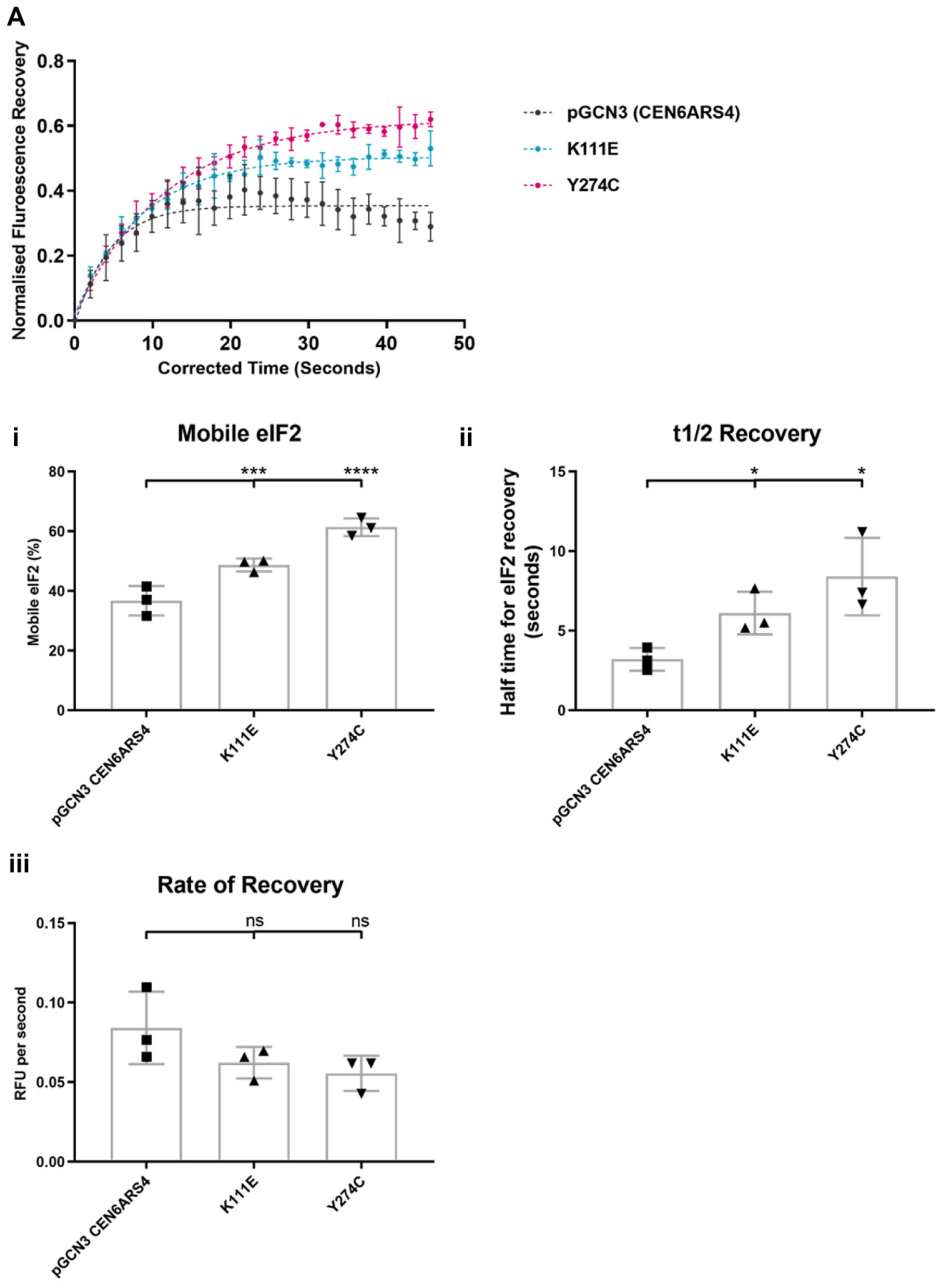
**Fluorescence recovery after photobleaching analysis of eIF2 cycling through eIF2B foci in the presence of vanishing white matter *gcn3* mutations reveal an increase in eIF2 recovery at a slower rate.** (*i*) normalized fluorescence recovery after photobleaching recovery curves for the strain ySC16 (*SUI2-yeGFPgcn3::LEU2*) expressing the vanishing white matter mutants Gcn3p^K11E^ (*p[GCN3K111EURA3CEN6ARS4]*), Gcn3p^Y274C^ (*p[GCN3Y274CURA3CEN6ARS4]*), and the low-copy WT plasmid *p[GCN3URA3CEN6ARS4]*. (*ii*) bar chart representing the percentage mobile eIF2 within the foci for each mutant. (*iii*) bar chart representing the T1/2 needed for eIF2 to fully recover. (*iv*) bar chart depicting the rate of eIF2 recovery. Data are representative of 25 cells per replicate, *n* = 3. Error bars are representative of SD. ∗*p* < 0.05, ∗∗*p* < 0.01, ∗∗∗*p* < 0.001, ∗∗∗∗*p* < 0.0001. eIF2B, eukaryotic initiation factor 2; ns, not significant.

In contrast, for the K111E and Y274C mutants, eIF2α–GFP did localize to eIF2B bodies and so, FRAP analysis was carried out to determine the impact of these mutations on the rate of eIF2 shuttling. One-phase association curves representing the recovery of eIF2 within eIF2B foci are shown in Figure 8*A*, whereas representative images from the various stages of FRAP are displayed in Fig. S5*E*. In the presence of the K111E mutation, mobile eIF2 increased by 12% (Fig. 8*B*) and the T_1/2_ recovery of eIF2 increased by 2.9 s, consistent with a decreased rate of recovery (Fig. 8*C*). Similarly, in the presence of the Y274C mutation, mobile eIF2 increased by 24.6% (Fig. 8*B*) and the T_1/2_ recovery of eIF2 increased by 5.2 s, again consistent with a decreased rate of recovery (Fig. 8*D*).

These results highlight that while VWM mutations may have a similar impact on the activity of eIF2B, they show contrasting effects on eIF2B localization. Currently, VWM pathophysiology is poorly understood largely because of limited correlation between the activity of mutant eIF2B and the severity of disease (33). These data highlight that VWM-causing mutations impact upon eIF2B localization, which may be contributing to the pathogenesis of these mutants.

## Discussion

In this study, we explored the significance of eIF2B body formation in terms of GEF activity and eIF2B regulation. Since the initial identification of eIF2B bodies, several publications have attempted to elucidate the driving factors behind the localization of eIF2B (24, 25, 26, 27, 28, 29, 30, 31). While these studies have highlighted many interesting observations, they have resulted in confounding conclusions. The conflicting reports raise the question of whether eIF2B bodies form under steady-state growth or just under nutrient-limiting conditions. We therefore considered whether the differences in the methodologies used to visualize eIF2B could be responsible for the observed differences in eIF2B body formation. The conflicting studies were carried out in different yeast strains. Several differences have been identified across these yeast strains particularly in their stress response phenotypes and also within genes that control ribosome biosynthesis (44, 45, 46). Distinct ribosomal subpopulations that differ in their protein or RNA components are known to differentially control translation and its regulation within the cell (47). We therefore hypothesized that strain differences may impact the formation of eIF2B bodies. In agreement with this, we found variation in the number of cells displaying eIF2B bodies across three different wildtype strains; with two strains harboring eIF2B bodies in approximately 50% of cells and the third strain presenting with a significantly lower percentage of cells harboring eIF2B bodies (Fig. 1*B*).

Another consideration for differences observed in eIF2B localization is whether the tag used to visualize eIF2B could be impacting on its localization. Here, we used a yeast-enhanced GFP tag to visualize eIF2B localization. GFP has a tendency to self-aggregate, and therefore, we wanted to ensure the eIF2B bodies we observed were not because of GFP aggregation. We individually tagged each of the five subunits of eIF2B and calculated the percentage of cells that contained eIF2B bodies. If the GFP tag was responsible for the aggregation of eIF2B into eIF2B bodies, we would expect to see the same percentage of cells with eIF2B bodies, regardless of the eIF2B subunit tagged. These experiments revealed that localization was not uniform across all subunits, suggesting that the eIF2B bodies we have observed are not because of aggregation of the GFP tag (Fig. 1*A*). This is further supported by our previous work where eIF2B bodies were observed using cyan fluorescent protein and YFP tags (24) and *via* immunolocalization studies using both endogenous and hemagglutinin-tagged subunits of eIF2B (24, 25).

**Figure 9:**
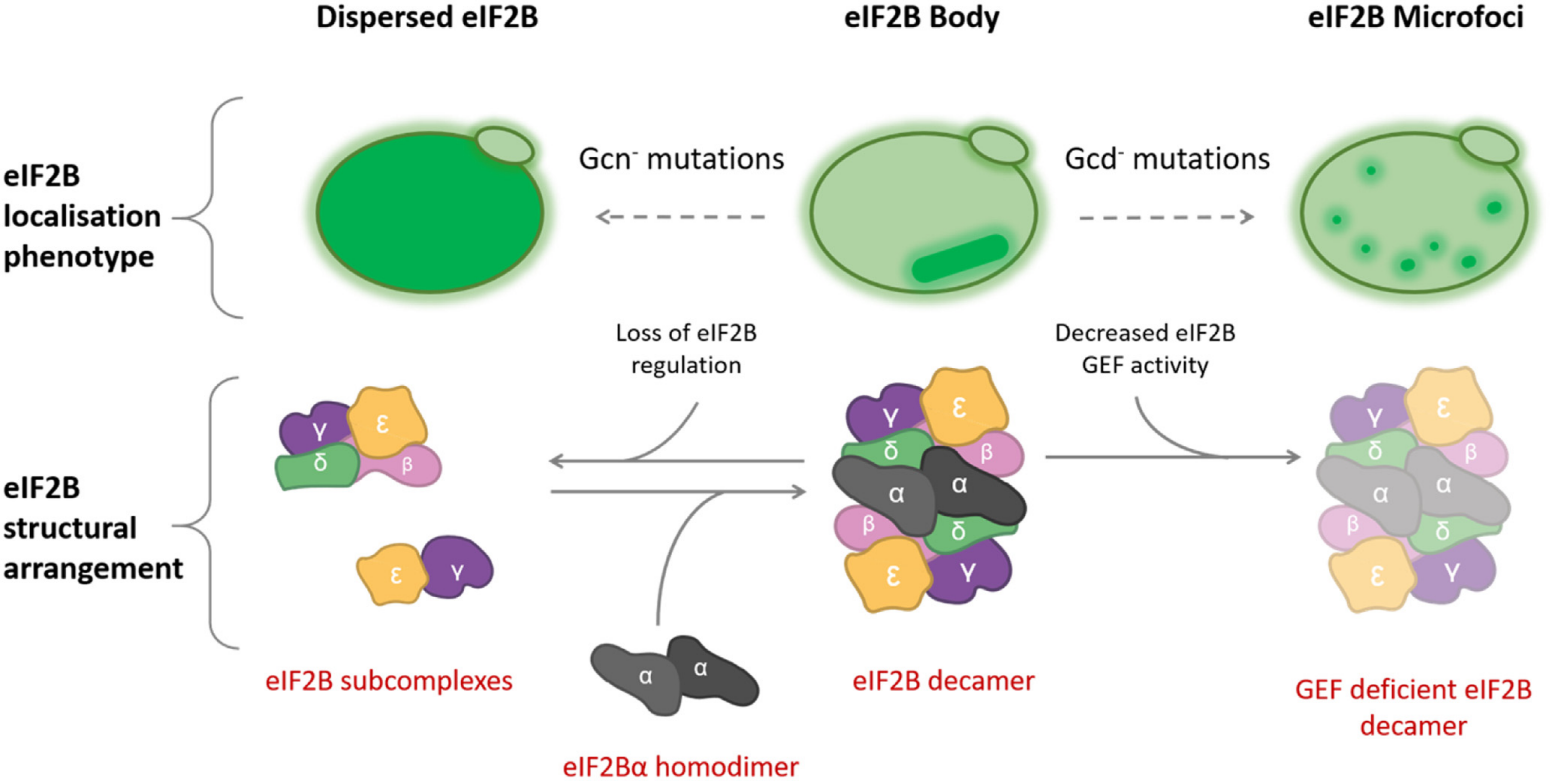
**Schematic of the relationship between the localization phenotype of eIF2B and its structure, activity, and regulation.** In yeast, eIF2B displays three distinct localization phenotypes: dispersed eIF2B, eIF2B bodies, or eIF2B microfoci. In its active decameric form, eIF2B localizes to eIF2B bodies. In the absence of eIF2Bα, this localization is lost, and a dispersed eIF2B localization phenotype is observed. eIF2B cannot form an octameric complex in the absence of eIF2Bα, and thus, we hypothesize eIF2B tetrameric and heterodimeric subcomplexes form. The dispersed eIF2B phenotype is mimicked by Gcn^−^ mutants suggesting a regulatory role for the eIF2B body. In addition, the localization of eIF2B to eIF2B bodies appears to enhance eIF2B GEF. Gcd^−^ mutants that decrease GEF activity disrupt eIF2B body localization and result in a neIF2B microfoci localization phenotype. The eIF2B microfoci have a decreased interaction with eIF2 and thus appear to have decreased eIF2B GEF activity. eIF2B, eukaryotic initiation factor 2B.

Interestingly, cells with GFP tagged eIF2Bα or δ showed a reduced percentage of cells containing eIF2B bodies, compared with the other three eIF2B subunits. This decrease could be due to the position of the tag. The C terminus of δ is important for heterodimerization with eIF2Bβ, whereas the C terminus of eIF2Bα is important for homodimerization (48, 49). Analysis of recent eIF2B structures reveals that the interface between eIF2Bα and δ is largely formed from the C terminus of both subunits (10, 11, 12, 13), and analytical centrifugation experiments have demonstrated that eIF2Bα is required to stabilize the eIF2B decamer, with eIF2B(βδγε) tetramers unable to dimerize in the absence of eIF2Bα homodimers (15). Therefore, it is likely that the addition of the GFP protein tag to the C terminus of either eIF2Bα or δ subunits would disrupt eIF2B decameric assembly. The decreased percentage of cells harboring eIF2B bodies suggests that eIF2B decameric assembly may promote the formation of eIF2B bodies. In fitting with this hypothesis, we previously highlighted that the localization of eIF2B bodies was dispersed in the presence of point mutations in eIF2Bα (26). Here, we have expanded on this and observed that deletion of the α subunit also leads to the complete dispersal of the eIF2B body (Fig. 2*A*). It is therefore likely that the eIF2B decamer must form before eIF2B is able to localize and multimerize to give eIF2B bodies, illustrated in our model of eIF2B body formation presented in Figure 9.

Although eIF2Bα is the only nonessential subunit of eIF2B in yeast, it does play critical roles in the regulation of translation initiation in response to stress (43). The dispersal of eIF2B bodies in the absence of eIF2Bα (Gcn3p) therefore suggests that the localization of eIF2B may have a regulatory role in translation as a response to cellular stress. This interpretation is consistent with previous work studying the regulation of eIF2B localization as a response to fusel alcohol stress where a decreased movement of eIF2B bodies within the cell was found to correlate with decreased translation (32). Furthermore, recent studies have suggested that eIF2B bodies form during cellular stress (28, 31). Although we observed different numbers of cells with eIF2B bodies in three different wildtype strains during steady-state growth, we were able to address the hypothesis that increased eIF2B body formation occurs during stress by monitoring localization under acute glucose starvation and the classical eIF2B-dependent stress, amino acid starvation (Fig. 1*B*). Although we did not see an increase in the number of cells displaying eIF2B bodies in the absence of glucose, we did see an increase when cells were subjected to amino acid starvation, suggesting that eIF2B-dependent stress can enhance the formation of eIF2B bodies.

A number of eIF2Bα mutations that abolish its regulatory function, thus rendering the cell unable to respond to stress, have been well characterized and are known as Gcn^−^ mutations. These Gcn^−^ mutations produced a phenotype where eIF2B was mostly dispersed throughout the cytoplasm (Fig. 4*Ai*). Interestingly, while these mutations did not impact on the expression level of eIF2Bα (Gcn3p), this phenotype mirrored the phenotype observed from *S. cerevisiae* lacking the α subunit (*gcn3Δ*), implying that Gcn^−^ mutations may completely abolish the α subunit from the decameric holocomplex (Fig. 9). In mammalian cells, eIF2B has been shown to form subcomplexes with reduced GEF activity in the absence of eIF2Bα (8, 29). The formation of these eIF2B subcomplexes has not been investigated in yeast; however, yeast expressing these Gcn^−^ mutations are viable, suggesting active complexes of eIF2B are present within the cells. Cryo-EM studies of eIF2B from *S. cerevisiae* localizes Gcn^−^ mutations to the eIF2ɑ-P interface as well as the eIF2Bα-β and eIF2Bα-δ interfaces (Fig. 3), suggesting that stable interactions between the regulatory subunits are required for localization (Fig. 4). This is consistent with recent analysis of the eIF2B cryo-EM structure, which indicates eIF2B bodies are formed by polymerization of intact eIF2B decamers (30). As the decameric conformation of eIF2B is required for eIF2B to recognize and interact with eIF2α-P (23), it is therefore plausible that eIF2B cannot sense stress in the presence of these Gcn^−^ mutants as a result of disruption to eIF2B assembly. Interestingly a series of *de novo* human *eIF2B1* variants have recently been identified in patients presenting with neonatal diabetes (50). Two of these novel *eIF2B1* mutations are equivalent residues to the Gcn^−^ residues analyzed in this work, and they all map to the surface region of eIF2B1 which interacts with phosphorylated eIF2α. This may imply that eIF2B localization may be an important feature of these regulatory mutations in this disease. Therefore, the Gcn-mutant analysis suggests that the eIF2B bodies are important for regulation.

We were also interested to investigate the importance of eIF2B bodies for eIF2B GEF activity itself. Gcd^−^ mutations, which decrease the catalytic activity of eIF2B, consistently produced a phenotype with multiple eIF2B foci, which we termed microfoci (Fig. 4*Aii*). However, Gcd^−^ mutants still respond to the phosphorylation of eIF2α suggesting that eIF2Bα is still present within eIF2B, and hence, the decameric structure is intact when these mutations are present (Fig. S2*B* and Fig. 9). Indeed, most of the Gcd^−^ Gcn3p mutations do not seem to directly impede interactions between eIF2Bα and eIF2Bβ/δ (Fig. 3). Instead, they are likely to affect the structural integrity of eIF2Bα subunit, but it is currently unclear how this would lead to reduced catalytic capacity of eIF2B. Interestingly, microfoci were previously observed when interactions between the very long fatty acids beta-keto-reductase Ifa38p and the catalytic subunits eIF2Bε/γ were abrogated (51). This suggests that other interactions are important for the complete localization of eIF2B to eIF2B bodies as well as its GEF activity. One exception observed in these studies was the AA 303//305Δ Gcd^−^ mutant, which did not significantly decrease the number of eIF2B bodies formed. Previous studies on this particular mutant highlighted that the loss of three amino acids at the extreme C-terminal domain upregulated the overall expression of *gcn3* 10-fold (40). Gcn3p appears to be integral for the formation of eIF2B bodies. If levels of Gcn3p are upregulated in 303//305Δ Gcd^−^ mutant cells, this overexpression of Gcn3p could partially rescue eIF2B body formation, thus cells displaying eIF2B bodies were observed in addition to the classical Gcd^−^ microfoci phenotype.

eIF2Bα VWM mutations were found to have diverse impacts on impact eIF2B body formation (Fig. 7*B*). VWM missense mutations, F240V and N209Y, displayed Gcn^−^ phenotypes, as determined by polysomal analysis (Fig. 7*A*). N209Y mutant cells displayed a similar dispersed eIF2B localization phenotype to the Gcn^−^ mutant strains. Biochemical analysis of this conserved mutant in human cells demonstrated that the mutant eIF2B complex was less sensitive to inhibition by eIF2α-P (35), supporting a role for eIF2B bodies in the regulation of eIF2B activity. In the F240V mutant, this dispersed phenotype was not observed, and instead eIF2B bodies formed, but in a reduced percentage of cells. Interestingly, eIF2 was not found to localize to these eIF2B bodies suggesting they are functionally impaired, perhaps contributing to the cells Gcn^−^ phenotype. For the other VWM mutants, a reporter analysis suggests that the there are no gross deficiencies in the eIF2B GEF activity (Fig. S4*B*); however, the V184D mutant harbored an increased percentage of cells with microfoci, and the eIF2B bodies present in K111E and Y274C mutant cells displayed a slower rate of eIF2 shuttling implying they have decreased GEF activity.

These data provide key insights into the formation of eIF2B bodies and their importance for eIF2B GEF activity and regulation within the cell. Under normal growth conditions, eIF2B bodies exist in a strain-dependent manner; however, in the presence of cellular stresses that target eIF2B activity through the phosphorylation of eIF2α, an increase in the percentage of cells harboring eIF2B bodies occurs independent of strain. These data highlight a regulatory role for eIF2B bodies during cellular stress. This is further highlighted by complete loss of eIF2B bodies in the presence of Gcn^−^ mutations, which abrogate the cells ability to respond to stress-induced eIF2αP. In addition to their regulatory role, the formation of eIF2B bodies appears to correlate with enhanced eIF2B catalytic activity. Gcd^−^ mutations, which decrease eIF2B GEF activity, disrupt eIF2B body formation resulting in cells exhibiting multiple smaller eIF2B-containing foci termed microfoci. The catalytic activity of these microfoci is reduced when compared with eIF2B bodies. VWM-causative point mutations also disrupted patterns of eIF2B localization, providing the first evidence that eIF2B localization may be linked to VWM pathophysiology. VWM disease has a wide clinical spectrum, and correlations between genotype and phenotype remain elusive. In certain cases, patients suffering the most severe VWM phenotypes harbor eIF2B mutations that biochemical studies have shown to have no impact on eIF2B complex formation or eIF2B activity (34). These data suggest that eIF2B modulation and regulation within eIF2B bodies may be a key facet to understanding the pathophysiology of VWM.

## Experimental procedures

### Strains construction and growth conditions

Yeast strain genotypes are displayed in Table 1 and derived from the W3031A yeast background strain with the exception of ySC, which is derived from BY4741 and yMK1180, which are derived from s288c yeast backgrounds. All strains are auxotrophic for specific amino acids or nucleobases. The strains were typically grown in rich YPD media (1% [w/v] yeast extract, 2% [w/v] bacto peptone, and 2% [w/v] glucose) or in minimal SCD (0.17% [w/v] yeast nitrogen base without amino acids, 0.5% [w/v] ammonium sulphate, and 2% [w/v] glucose) at 30 °C (52), supplemented with specific dropouts (Formedium) depending on the genotype of the strain. In liquid media, strains were incubated at 30 °C with agitation, whereas growth on solid media, YPD or SCD was supplemented with 2% (w/v) agar. Nutrient starvation was performed by the removal of all amino acids for 30 min, and carbon source starvation was performed by the removal of glucose for 10 min.

**Table 1:** Yeast strains used within this study

Strain	Genotype	Source
yMK880	*Matα*, *ADE2*, *his3-11,15*, *leu2-3,112*, *trp1-1*, *ura3-1*, *can1-100*, *GCD1-P180-yeGFP::G418*	(24)
yMK883	*Matα*, *ADE2*, *his3-11,15*, *leu2-3,112*, *trp1-1*, *ura3-1*, *can1-100*, *GCD1-P180*, *SUI2-yeGFP::G418*	(24)
yMK1402	*Matα*, *ADE2*, *his3-11,15*, *leu2-3,112*, *trp1-1*, *ura3-1*, *can1-100*, *GCD1-P180-yeGFP::G418*, *gcn3::LEU2*	(25)
ySC37	*Matα*, *ADE2*, *his3-11,15*, *leu2-3,112*, *trp1-1*, *ura3-1*, *can1-100*, *GCD1-P180-yeGFP::G418*, *gcn3::LEU2*, *p[GCN3 URA3 2μ]*	This study
ySC38	*Matα*, *ADE2*, *his3-11,15*, *leu2-3,112*, *trp1-1*, *ura3-1*, *can1-100*, *GCD1-P180-yeGFP::G418*, *gcn3::LEU2, p[GCN3-T41A URA3 2μ]*	This study
ySC39	*Matα*, *ADE2*, *his3-11,15*, *leu2-3,112*, *trp1-1*, *ura3-1*, *can1-100*, *GCD1-P180-yeGFP::G418*, *gcn3::LEU2*, *p[GCN3-E44V URA3 2μ]*	This study
ySC40	*Matα*, *ADE2*, *his3-11,15*, *leu2-3,112*, *trp1-1*, *ura3-1*, *can1-100*, *GCD1-P180-yeGFP::G418*, *gcn3::LEU2*, *p[GCN3-E44K URA3 2μ]*	This study
ySC41	*Matα*, *ADE2*, *his3-11,15*, *leu2-3,112*, *trp1-1*, *ura3-1*, *can1-100*, *GCD1-P180-yeGFP::G418*, *gcn3::LEU2*, *p[GCN3-F73L URA3 2μ]*	This study
ySC42	*Matα*, *ADE2*, *his3-11,15*, *leu2-3,112*, *trp1-1*, *ura3-1*, *can1-100*, *GCD1-P180-yeGFP::G418*, *gcn3::LEU2*, *p[GCN3-N80D URA3 2μ]*	This study
ySC43	*Matα*, *ADE2*, *his3-11,15*, *leu2-3,112*, *trp1-1*, *ura3-1*, *can1-100*, *GCD1-P180-yeGFP::G418*, *gcn3::LEU2*, *p[GCN3-F240I URA3 2μ]*	This study
ySC44	*Matα*, *ADE2*, *his3-11,15*, *leu2-3,112*, *trp1-1*, *ura3-1*, *can1-100*, *GCD1-P180-yeGFP::G418*, *gcn3::LEU2*, *p[GCN3-T291P URA3 2μ]*	This study
ySC45	*Matα*, *ADE2*, *his3-11,15*, *leu2-3,112*, *trp1-1*, *ura3-1*, *can1-100*, *GCD1-P180-yeGFP::G418*, *gcn3::LEU2*, *p[GCN3-S293P URA3 2μ]*	This study
ySC46	*Matα*, *ADE2*, *his3-11,15*, *leu2-3,112*, *trp1-1*, *ura3-1*, *can1-100*, *GCD1-P180-yeGFP::G418*, *gcn3::LEU2*, *p[GCN3 URA3 CEN]*	This study
ySC47	*Matα*, *ADE2*, *his3-11,15*, *leu2-3,112*, *trp1-1*, *ura3-1*, *can1-100*, *GCD1-P180-yeGFP::G418*, *gcn3::LEU2*, *p[GCN3-A25V, A26V URA3 CEN]*	This study
ySC48	*Matα*, *ADE2*, *his3-11,15*, *leu2-3,112*, *trp1-1*, *ura3-1*, *can1-100*, *GCD1-P180-yeGFP::G418*, *gcn3::LEU2*, *p[GCN3-A26T URA3 CEN]*	This study
ySC49	*Matα*, *ADE2*, *his3-11,15*, *leu2-3,112*, *trp1-1*, *ura3-1*, *can1-100*, *GCD1-P180-yeGFP::G418*, *gcn3::LEU2*, *p[GCN3-R104K URA3 CEN]*	This study
ySC50	*Matα*, *ADE2*, *his3-11,15*, *leu2-3,112*, *trp1-1*, *ura3-1*, *can1-100*, *GCD1-P180-yeGFP::G418*, *gcn3::LEU2*, *p[GCN3-V295F URA3 CEN]*	This study
ySC51	*Matα*, *ADE2*, *his3-11,15*, *leu2-3,112*, *trp1-1*, *ura3-1*, *can1-100*, *GCD1-P180-yeGFP::G418*, *gcn3::LEU2*, *p[GCN3-D71N URA3 CEN]*	This study
ySC52	*Matα*, *ADE2*, *his3-11,15*, *leu2-3,112*, *trp1-1*, *ura3-1*, *can1-100*, *GCD1-P180-yeGFP::G418*, *gcn3::LEU2*, *p[GCN3-E199K URA3 CEN]*	This study
ySC53	*Matα*, *ADE2*, *his3-11,15*, *leu2-3,112*, *trp1-1*, *ura3-1*, *can1-100*, *GCD1-P180-yeGFP::G418*, *gcn3::LEU2*, *p[GCN3-AA303–305Δ URA3 CEN]*	This study
ySC54	*Matα*, *ADE2*, *his3-11,15*, *leu2-3,112*, *trp1-1*, *ura3-1*, *can1-100*, *GCD1-P180-yeGFP::G418*, *gcn3::LEU2*, *p[GCN3 URA3 CEN4ARS6]*	This study
ySC55	*Matα*, *ADE2*, *his3-11,15*, *leu2-3,112*, *trp1-1*, *ura3-1*, *can1-100*, *GCD1-P180-yeGFP::G418*, *gcn3::LEU2*, *p[GCN3-K111E URA3 CEN4ARS6]*	This study
ySC56	*Mat α*, *ADE2*, *his3-11,15*, *leu2-3,112*, *trp1-1*, *ura3-1*, *can1-100*, *GCD1-P180-yeGFP::G418*, *gcn3::LEU2*, *p[GCN3-V184D URA3 CEN4ARS6]*	This study
ySC57	*Mat α*, *ADE2*, *his3-11,15*, *leu2-3,112*, *trp1-1*, *ura3-1*, *can1-100*, *GCD1-P180-yeGFP::G418*, *gcn3::LEU2*, *p[GCN3-N209Y URA3 CEN4ARS6]*	This study
ySC58	*Mat α*, *ADE2*, *his3-11,15*, *leu2-3,112*, *trp1-1*, *ura3-1*, *can1-100*, *GCD1-P180-yeGFP::G418*, *gcn3::LEU2*, *p[GCN3-F240V URA3 CEN4ARS6]*	This study
ySC59	*Mat α*, *ADE2*, *his3-11,15*, *leu2-3,112*, *trp1-1*, *ura3-1*, *can1-100*, *GCD1-P180-yeGFP::G418*, *gcn3::LEU2*, *p[GCN3-Y274C URA3 CEN4ARS6]*	This study
ySC16	*Mat α*, *ADE2*, *his3-11,15*, *leu2-3,112*, *trp1-1*, *ura3-1*, *can1-100*, *GCD1-P180*, *SUI2-GFP::G418*, *gcn3::LEU2*	This study
ySC61	*Mat α*, *ADE2*, *his3-11,15*, *leu2-3,112*, *trp1-1*, *ura3-1*, *can1-100*, *GCD1-P180*, *SUI2-GFP::G418*, *gcn3::LEU2*, *p[GCN3 URA3 CEN]*	This study
ySC62	*Mat α*, *ADE2*, *his3-11,15*, *leu2-3,112*, *trp1-1*, *ura3-1*, *can1-100*, *GCD1-P180*, *SUI2-GFP::G418*, *gcn3::LEU2*, *p[GCN3-A25V,A26V URA3 CEN]*	This study
ySC67	*Mat α*, *ADE2*, *his3-11,15*, *leu2-3,112*, *trp1-1*, *ura3-1*, *can1-100*, *GCD1-P180*, *SUI2-GFP::G418*, *gcn3::LEU2*, *p[GCN3-E199K URA3 CEN]*	This study
ySC68	*Mat α*, *ADE2*, *his3-11,15*, *leu2-3,112*, *trp1-1*, *ura3-1*, *can1-100*, *GCD1-P180*, *SUI2-GFP::G418*, *gcn3::LEU2*, *p[GCN3-AA303-305Δ URA3 CEN]*	This study
ySC9	*MATa his3Δ1 leu2Δ0 met15Δ0 ura3Δ0 GCD1-yeGFP::HygR*	This study
yMK1180	*MATα leu2-3*, *112*, *ura3-52::[HIS4-lacZ ura3-52] ino1, gcd6Δ, gcn2Δ::hisG, GCD1-yeGFP::G418, p[GCD6 CEN6 LEU2]*	
ySC91	*Mat α, ADE2, his3-11,15, leu2-3,112, trp1-1, ura3-1, can1-100, GCD1-P180-yeGFP::G418, GCN4-LacZ-TRP1, gcn3::HIS3 p[GCN3 URA3 CEN6ARS4]*	This study
ySC92	*Matα, ADE2, his3-11,15, leu2-3,112, trp1-1, ura3-1, can1-100, GCD1-P180-yeGFP::G418, GCN4-LacZ-TRP1, gcn3::HIS3 p[gcn3 K111E URA3 CEN6ARS4]*	This study
ySC93	*Matα, ADE2, his3-11,15, leu2-3,112, trp1-1, ura3-1, can1-100, GCD1-P180-yeGFP::G418, GCN4-LacZ-TRP1, gcn3::HIS3 p[gcn3 V184D URA3 CEN6ARS4]*	This study
ySC94	*Matα, ADE2, his3-11,15, leu2-3,112, trp1-1, ura3-1, can1-100, GCD1-P180-yeGFP::G418, GCN4-LacZ-TRP1, gcn3::HIS3 p[gcn3 N209Y URA3 CEN6ARS4]*	This study
ySC95	*Matα, ADE2, his3-11,15, leu2-3,112, trp1-1, ura3-1, can1-100, GCD1-P180-yeGFP::G418, GCN4-LacZ-TRP1, gcn3::HIS3 p[gcn3 F240V URA3 CEN6ARS4]*	This study
ySC96	*Matα, ADE2, his3-11,15, leu2-3,112, trp1-1, ura3-1, can1-100, GCD1-P180-yeGFP::G418, GCN4-LacZ-TRP1, gcn3::HIS3 p[gcn3 Y274C URA3 CEN6ARS4]*	This study

### Plasmids used in this study

The plasmids used in this study are shown in Table 2. A number of plasmids, denoted pAV, were kindly gifted by Prof. G. Pavitt (The University of Manchester).

**Table 2:** Plasmids used within this study

Name	Genotype	Source
pSC116	*p[GCN3 K111E URA3 CEN6ARS4]*	This study
pSC117	*p[GCN3 V184D URA3 CEN6ARS4]*	This study
pSC118	*p[GCN3 F240V URA3 CEN6ARS4]*	This study
pSC119	*p[GCN3 Y274C URA3 CEN6ARS4]*	This study
pAV1108	*p[GCN3(T41A) URA3 2μ]*	(40)
pAV1109	*p[GCN3(E44V)URA3 2μ]*	(40)
pAV1110	*p[GCN3(E44K)URA3 2μ]*	(40)
pAV1111	*p[GCN3(F73L) URA3 2μ]*	(40)
pAV1112	*p[GCN3(N80D) URA3 2μ]*	(40)
pAV1113	*p[GCN3(F240I) URA3 2μ]*	(40)
pAV1115	*pGCN3 (T291P) URA3 2μ]*	(40)
pAV1116	*p[GCN3 (S293R) URA3 2μ]*	(40)
pAV1117	*p[GCN3 URA3 2μ]*	(40)
pAV1170	*p[GCN3 URA3 CEN]*	(40)
pAV1239	*p[GCN3(AA2526VV) URA3 CEN]*	(40)
pAV1240	*p[GCN3(A26T) URA3 CEN]*	(40)
pAV1241	*p[GCN3(R104K) URA3 CEN]*	(40)
pAV1242	*p[GCN3(V295F) URA3 CEN]*	(40)
pAV1243	*p[GCN3(D71N) URA3 CEN]*	(40)
pAV1244	*p[GCN3(E199K) URA3 CEN]*	(40)
pAV1268	*p[GCN3(303//305Δ) URA3 CEN]*	(40)
pAV1729	*p[GCN4 leader-lacZ-TRP1]*	(40)
pAV1769	*p[GCN3 URA3 CEN6 ARS4]*	(43)
pAV1778	*p[GCN3(N209Y) URA3 CEN6 ARS4]*	(43)

### Site-directed mutagenesis

Site-directed mutagenesis was performed using the QuikChange II XL kit (Agilent) as instructed by the manufacturers. Plasmid DNA was isolated from multiple independent transformants, and plasmids were Sanger sequenced to confirm the desired mutation had been generated.

### Western blot analysis

Protein extracts were generated from yeast cultures grown to an absorbance of 0.6 at 600 nm. All cells were lysed, and protein samples were prepared, electrophoretically separated, and subjected to immunoblot analysis as described previously (24). Western blotting was carried out as previously described (24) using the following antibodies: eIF2Bα/Gcn3p (a kind gift from Prof. G. Pavitt, The University of Manchester, UK) at 1:500 dilution and Pab1p (a kind gift from Prof. M. Ashe, The University of Manchester, UK) at 1:5000 dilution. Primary antibodies were detected using Goat anti-rabbit IRDye 680RD P/N 925-68071 and goat antimouse IRDye 800CW P/N 925-32210 (Licor), respectively.

### Assays of GCN4–lacZ reporter expression

Standard methods for measuring the β-galactosidase activity for strains bearing *GCN4–lacZ* fusions were used (53). β-Galactosidase levels are expressed as nanomoles of *o*-nitrophenol β-D-galactopyranoside hydrolyzed per min/μg of total protein.

### Live-cell imaging and quantification of eIF2B localization

Strains were grown at 30 °C until they reached an absorbance of 0.6 at 600 nm. Cultures were placed on a 1% (w/v) poly-L-lysine–coated slide (ThermoFisher, UK) and visualized on a Zeiss LSM 510 confocal microscope using a 63× plan-apochromat oil objective lens. To image GFP, an argon laser (488 nm) was typically used with a maximum output of 25 mW at 55% laser capacity. Images were analyzed either using Zeiss 2009 software or the National Institutes of Health (NIH) ImageJ software. For each strain/mutant analyzed, the localization of eIF2B was assessed for 100 cells per replicate (unless otherwise stated), and three independent replicates were performed. Three different localization patterns were observed for eIF2B. The localization pattern was defined as an eIF2B body, in cells where one large, commonly filamentous, structure could be observed. In cells where eIF2B localized to multiple smaller punctate foci, these foci were defined as eIF2B microfoci. In cells where no eIF2B foci were visible and instead fluorescence was evenly distributed across the cytoplasm, the localization phenotype of eIF2B was referred to as dispersed.

### FRAP

FRAP was performed to measure the shuttling of eIF2α–GTP through eIF2B foci as previously described (24). Cytoplasmic foci were imaged and bleached using the argon laser at full capacity. Following the prebleach and bleach steps, the recovery of eIF2B into the cytoplasm was followed by taking iterative images every 1.8 s for 25 cycles. About 25 cells were analyzed for each replicate.

Fluorescence recovery was normalized to the total fluorescence of the cell. Background fluorescence was also measured and subtracted from fluorescence recovery. Normalized data were fitted to a one-phase association curve to find mobile eIF2 and half-time recovery. A rate of recovery was calculated from the one-phase association curves by dividing the plateau by the rate constant (*k*). The eIF2 content of cytoplasmic foci was determined using NIH ImageJ software.

### Analysis of ribosome distribution on sucrose gradients

Yeast cultures were grown to an absorbance of 0.6 at 600 nm and harvested by centrifugation. When cells were subjected to nutritional stress (*e.g.*, amino acid or glucose starvation), cultures were split into two 50 ml cultures, centrifuged, and resuspended in media either with or without amino acids or glucose, as described previously. Cells were lysed in polyribosomal buffer containing 100 μg/ml cycloheximide, and 2.5 units of extracts at an absorbance at 260 nm were layered onto 15 to 50% sucrose gradients. Sucrose gradients were poured as previously described (24). The gradients were sedimented *via* ultracentrifugation at 40,000 rpm using a Th-641 swing-out rotor in a Sorvall WX Ultracentrifuge or 2.5 h. Monosome and polysome peaks were quantified using the NIH ImageJ software (http://rsb.info.nih.gov/ij/).

### Statistical analysis

To determine statistical significance between different groups within each data set, a Shapiro–Wilk test was performed to test for normality. All data presented were considered nonparametric, and therefore, individual groups were compared with each other using the Kruskal–Wallis test followed by a Conover Inman post hoc test.

### Structural analysis of eIF2Bα mutations

PyMOL was used to produce eIF2B structural representations using cryo-EM structure data derived from *S. cerevisiae*, Protein Data Bank ID: 6i3m and 6i7t (10). The structure of the eIF2B complex is shown in ribbon representation with individual subunits colored and labeled. The eIF2Bα homodimer is shown in surface representation with residues residing within the interface between the eIF2Bα subunits, and either eIF2B subunits or the alpha subunit of eIF2 are color coded. eIF2Bα mutant residues were identified and labeled with proximal residues from other eIF2B subunits also labeled for visual representation.

## Data availability

All data are included within the article.

## Conflict of interest

The authors declare that they have no conflicts of interest with the contents of this article.
