# Supplementary material for: Mutational analysis of the alpha subunit of eIF2B provides insights into the role of eIF2B bodies in translational control and VWM disease
Source: J Biol Chem. 2021 Jan 30;296:100207. doi: 10.1074/jbc.RA120.014956 (PMC7948505; doi:10.1074/jbc.RA120.014956)
Supplement: Figures S1–S5 [file mmc1.pdf]

Supporting Information

Mutational analysis of the alpha subunit of eIF2B provides insights into the role of eIF2B bodies in translational control and VWM disease

Karl Norris[1,#`], Rachel E. Hodgson[1`], Tawni Dornelles[2], K. Elizabeth Allen[1], Ben M. Abell[1], Mark P. Ashe[2] and Susan G. Campbell[1*].

[1]Biomolecular Sciences Research Centre, Sheffield Hallam University, Howard Street, Sheffield, S1 1WB, UK. [2]Division of Molecular and Cellular Function, Faculty of Biology, Medicine and Health, The University of Manchester, Manchester, M13 9PTUK.

[#]Present Address: School of Cellular and Molecular Biology, Faculty of Biological Sciences, University of Leeds, Leeds LS2 9JT UK

` both authors contributed equally to this work.

* To whom correspondence should be addressed: Susan G. Campbell, Biomolecular Sciences Research Centre, Sheffield Hallam University, Howard Street, Sheffield, S1 1WB, UK; E-mail: Email: susan.campbell@shu.ac.uk

Running title: eIF2Bα contributes to the formation of eIF2B bodies

Keywords: eIF2B, eIF2, translation initiation, VWM

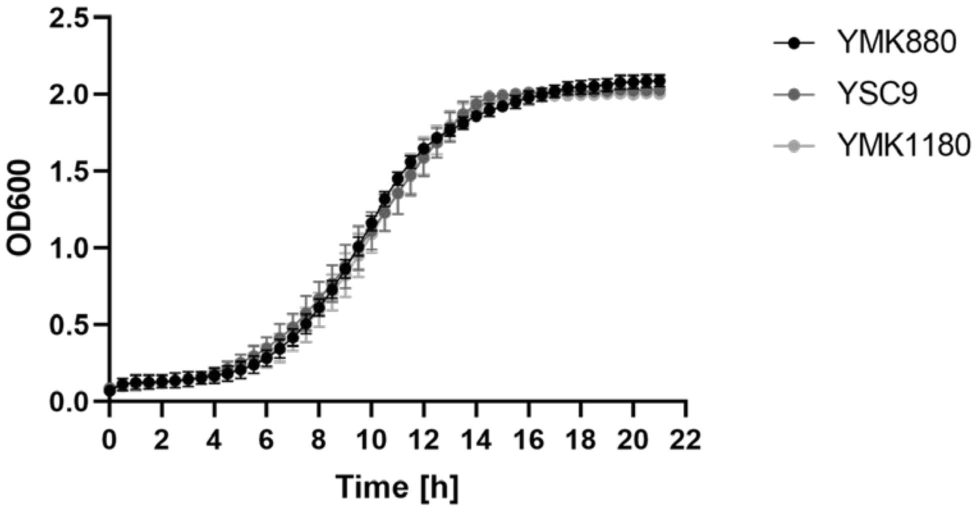

Figure S1 – The presence of the eIF2B fusion protein does not influence the growth of different lab strains of *S. cerevisiae*. Growth curve derived from three biological replicate cultures of the 3 *S. cerevisiae* strains containing the C-terminally GFP-tagged *GCD1* (eIF2Bγ), W303-1A (yMK880), BY4741 (ySC9) and S288c (yMK1180). For these growth curves, three single colonies were each grown simultaneously under identical nutritional and growth conditions.

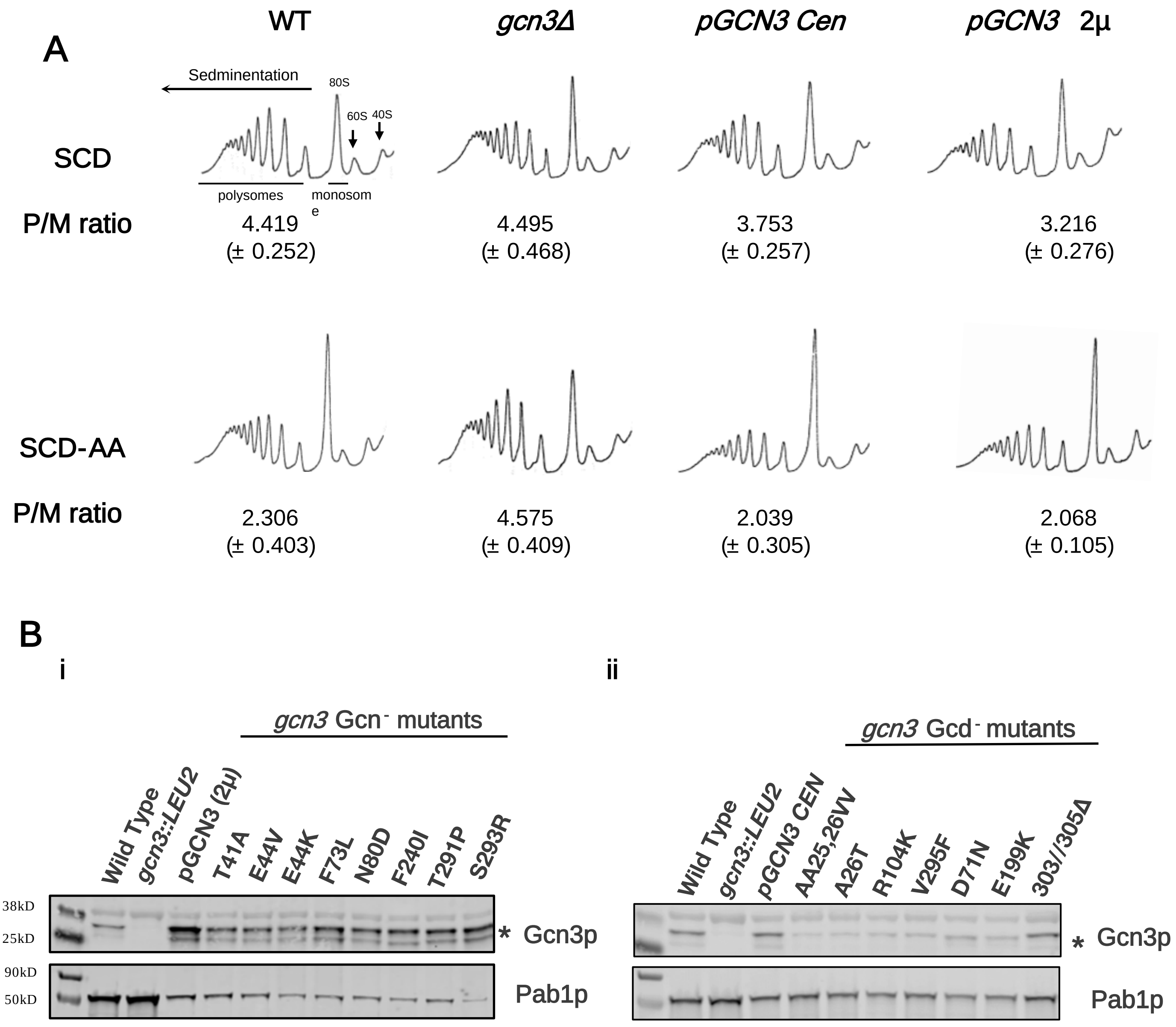

Figure S2 – Gcn3p is required for translational regulation during amino acid starvation. (A) Polysome analysis of the strain yMK1402 bearing (*GCD1-yeGFP gcn3::LEU2)* transformed with either a low copy Cen plasmid (pAV1170) or a high copy 2µ plasmid (pAV1117) containing WT *GCN3*. The strains were grown in selective media and incubated either in fresh media (SCD) or amino acid–free media (SCD-AA) or 15 min at 30°C. Polysome/ monosome ratios were calculated from measuring the area under the polysome peaks and dividing by the monosome peak area. Polysome analysis was as described in *Materials and Methods*. (B) Western blots were performed on protein extracts prepared from yMK880 and the strain yMK1402 (GCD1-yeGFP gcn3::LEU2) containing a series of (i) Gcn^- mutants [pAV1108–13, 15 and 16] and (ii) Gcd^- mutants [pAV1238-44 and 68]. The westerns were probed with antibodies to anti Gcn3p and anti Pab1p used as a loading control

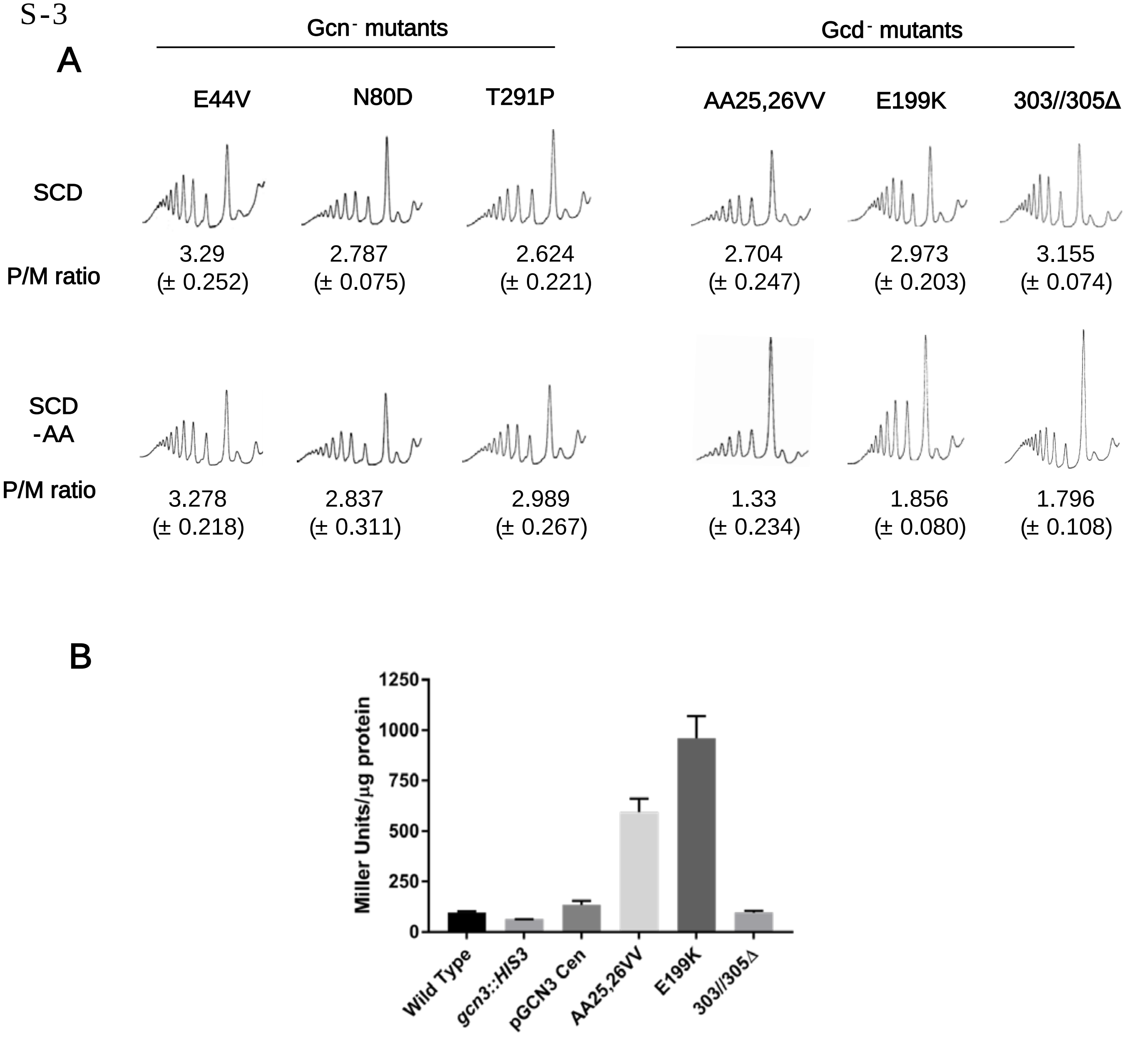

Figure S3 – Gcn3p Gcn^{-}/Gcd^{-} mutants are unable to respond to amino acid stress while some gcd^{-} mutants derepress *GCN4* and constitutively instigate a stress response. (A) Polysome profiling of GCD1-yeGFP gcn3::LEU2 (yMK1402) exogenously expressing Gcn^{-} or Gcd^{-} mutations (Gcn-: pAV1108 – 13, 15 and 16. Gcd-: pAV1238-44 and 68). The Gcn^{-} mutations were previously generated using a high copy 2μ GCN3 plasmid (pAV1117) while the Gcd^{-} mutations were generated using a low copy CEN GCN3 plasmid (pAV1170). The strains were grown in selective media and incubated either in fresh media (SCD) or amino acid–free media (SCD-AA) or 15 min at 30°C. Polysome/ monosome ratios were calculated from measuring the area under the polysome peaks and dividing by the monosome peak area. Polysome analysis was as described in *Materials and Methods*. (B) GCN4-LacZ assay was performed on the strain ySC86 (*GCN4-LacZ, gcn3::HIS3) expressing wt* Gcn3p *(p[GCN3 URA3 CEN])*, Gcn3p^{AA25,26VV} *(p[GCN3(AA2526VV) URA3 CEN])*, Gcn3p^{E199K} *(p[GCN3(E199K) URA3 CEN]) or* Gcn3p$^{303//305\Delta}$ *(p[GCN3(303//305Δ)])*. Cultures were diluted to 0.2 OD_{600} and incubated at 30°C with shaking until exponential growth was reached. Total protein was extracted and the β-galactosidase activity was measured to assess Gcn4p expression. Protein concentration was determined via BCA assay. n=3, error bars are representative of SD.

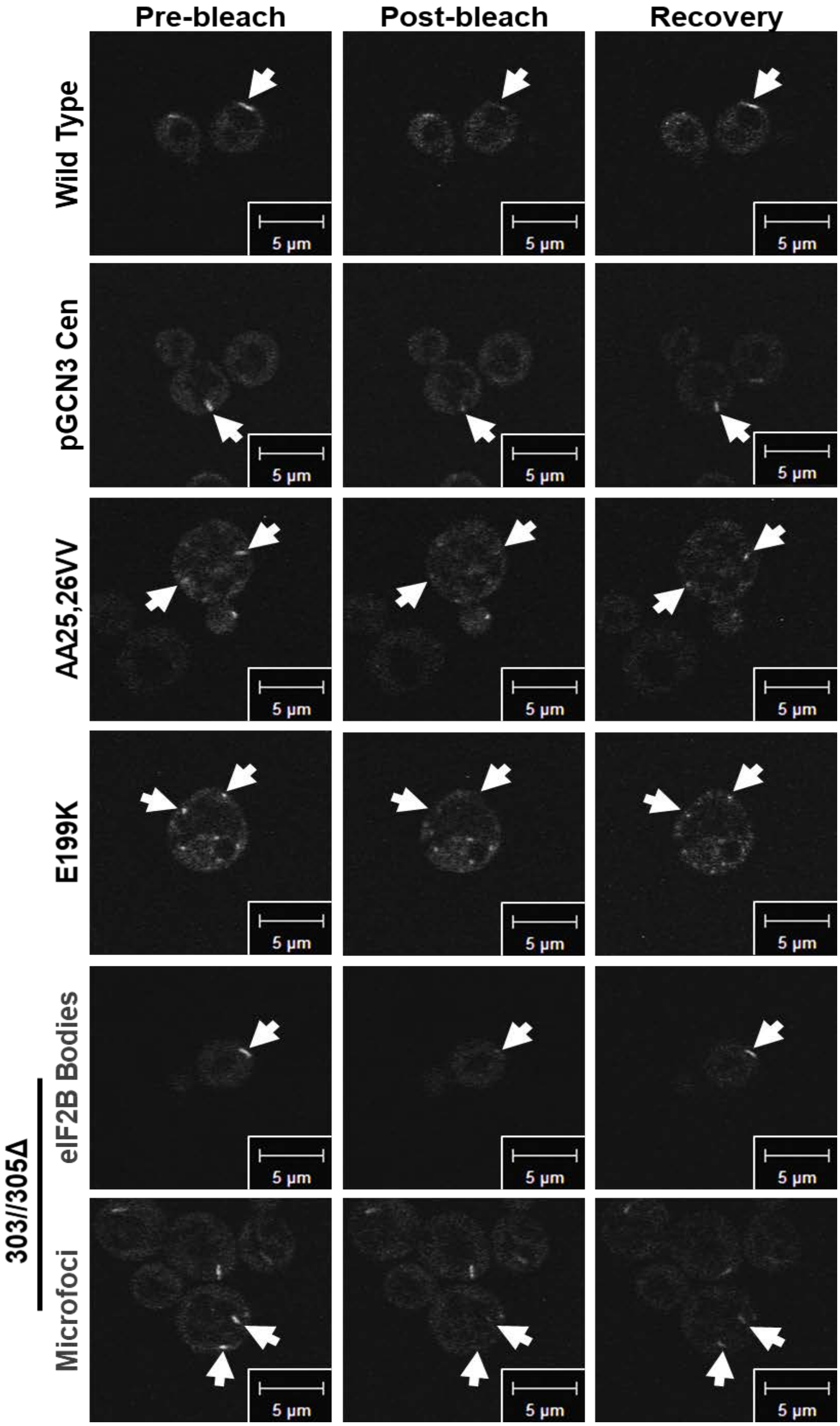

Figure S4 – Representative images of FRAP stages for Gcn3p Gcd^{-} mutants *(see figure 5).* Cells were grown to log phase and confocal microscopy was used to FRAP analysis. Representative images of the different stages of the FRAP experiment (pre-bleach, bleach and recovery) are shown for the strain ySC16 (*SUI2-yeGFP gcn3::LEU2*) expressing WT Gcn3p (p[*GCN3 URA3 CEN6ARS4*]), Gcn3p^{K11E} (p[*GCN3-K11E URA3CEN6ARS4*]), Gcn3p^{AA25,26VV} (p[*GCN3-AA25,26VVURA3 CEN]) or* Gcn3p$^{303//305\Delta}$ *(p[GCN3-303//305 URA3 CEN]).*

S-5

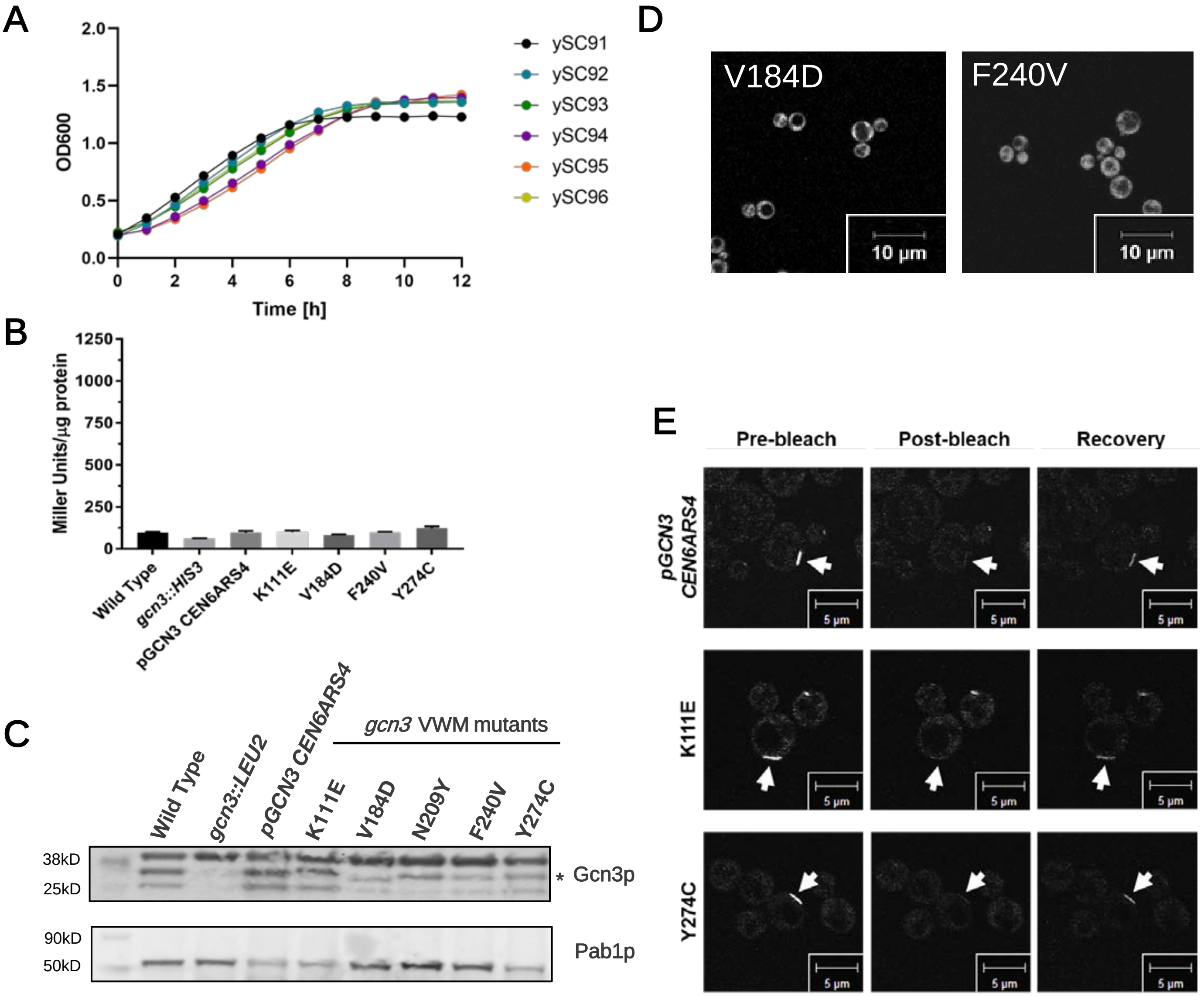

Figure S5 – Representative Growth curves, GCN4-LacZ assays and FRAP images for VWM mutants. (A) Growth curve derived from three biological replicate cultures of the strain yMK1402 (*GCD1-yeGFP gcn3::LEU2*) expressing gcn3 VWM mutants, Gcn3p^{K11E} (*ySC92[GCN3 K111E URA3 CEN6ARS4]),* Gcn3p^{V184D} *(ySC93[GCN3 V184D URA3 CEN6ARS4]),* Gcn3p^{N209Y} *(ySC94[GCN3 N209Y URA3 CEN6ARS4]),* Gcn3p^{F240V} *(ySC95[GCN3 F240V URA3 CEN6ARS4]),* Gcn3p^{Y274C} *(ySC96[GCN3 Y274C URA3 CEN6ARS4])* and the low copy WT plasmid *ySC91[GCN3 URA3 CEN6 ARS4]*. For these growth curves, three single colonies were each grown simultaneously under identical nutritional and growth conditions. (B) GCN4-LacZ assay of four VWM gcn3 mutants exogenously expressed in a GCN4-LacZ, gcn3::HIS3 (ySC86) strain. Cultures were diluted to 0.2 OD600 and incubated at 30°C with shaking until exponential growth was reached. Total protein was extracted and the β-galactosidase activity was measured to assess Gcn4p expression. Protein concentration was determined via BCA assay. n=3, error bars are representative of SD (n=3). C)Western blot to assess levels of Gcn3p in the different strains lacking Gcn3p or expressing Gcn3p endogenously or exogenously. D) Exponential cultures of ySC9 (*SUI2-GFP gcn3::LEU2*) expressing either Gcn3p^{V164D} (p[*GCN3-V164D URA3 CEN6]) or* Gcn3p^{F240V} *(p[GCN3*-F240V URA3 CEN6]) VWM mutants were live-cell imaged via confocal microscopy. (E) Live-cell images of the different FRAP stages are shown for the strain ySC16 (*SUI2-yeGFP gcn3::LEU2*) containing p[W*T GCN3 URA3 CEN6ARS4*], p[*GCN3-K11E URA3CEN6ARS4*] or p[*GCN3-Y274C URA3CEN6ARS4*].
